# Defective Neuronal Positioning Correlates With Aberrant Motor Circuit Function in Zebrafish

**DOI:** 10.3389/fncir.2021.690475

**Published:** 2021-06-24

**Authors:** Emilia Asante, Devynn Hummel, Suman Gurung, Yasmin M. Kassim, Noor Al-Shakarji, Kannappan Palaniappan, Vinoth Sittaramane, Anand Chandrasekhar

**Affiliations:** ^1^Division of Biological Sciences and Bond Life Sciences Center, University of Missouri, Columbia, MO, United States; ^2^Department of Pathology and Cell Biology, USF Health Heart Institute, University of South Florida, Tampa, Florida, FL, United States; ^3^Computational Imaging and VisAnalysis (CIVA) Lab, Department of Electrical Engineering and Computer Science, University of Missouri, Columbia, MO, United States; ^4^Department of Biology, Georgia Southern University, Statesboro, GA, United States

**Keywords:** jaw movement, food intake, behavior, neuronal migration, axon guidance, zebrafish, neural circuits, facial branchiomotor neuron

## Abstract

Precise positioning of neurons resulting from cell division and migration during development is critical for normal brain function. Disruption of neuronal migration can cause a myriad of neurological disorders. To investigate the functional consequences of defective neuronal positioning on circuit function, we studied a zebrafish *frizzled3a* (*fzd3a*) loss-of-function mutant *off-limits* (*olt*) where the facial branchiomotor (FBM) neurons fail to migrate out of their birthplace. A jaw movement assay, which measures the opening of the zebrafish jaw (gape), showed that the frequency of gape events, but not their amplitude, was decreased in *olt* mutants. Consistent with this, a larval feeding assay revealed decreased food intake in *olt* mutants, indicating that the FBM circuit in mutants generates defective functional outputs. We tested various mechanisms that could generate defective functional outputs in mutants. While *fzd3a* is ubiquitously expressed in neural and non-neural tissues, jaw cartilage and muscle developed normally in *olt* mutants, and muscle function also appeared to be unaffected. Although FBM neurons were mispositioned in *olt* mutants, axon pathfinding to jaw muscles was unaffected. Moreover, neuromuscular junctions established by FBM neurons on jaw muscles were similar between wildtype siblings and *olt* mutants. Interestingly, motor axons innervating the interhyoideus jaw muscle were frequently defasciculated in *olt* mutants. Furthermore, GCaMP imaging revealed that mutant FBM neurons were less active than their wildtype counterparts. These data show that aberrant positioning of FBM neurons in *olt* mutants is correlated with subtle defects in fasciculation and neuronal activity, potentially generating defective functional outputs.

## Introduction

The complex architecture of the vertebrate brain, especially the mammalian brain, is generated in part by the coordinated and orchestrated migration of neurons during development. Neuronal progenitors often migrate extensively using two modes to disperse throughout the central nervous system, radial migration, and tangential migration (de Rouvroit and Goffinet, 2001; Marín et al., 2010; Sun et al., 2010). These events are critical developmental processes essential for precise neuronal positioning and establishing functional neural circuits (Gilmore and Herrup, 1997; Tau and Peterson, 2010). Failure of neuronal migration can lead to brain malformations associated with neurological conditions such as lissencephaly, epilepsy, and autism spectrum disorders (Copp and Harding, 1999; Gleeson and Walsh, 2000; Manent and LoTurco, 2013; Pan et al., 2019). In type 1 lissencephaly, characterized by a lack of gyrification, pyramidal projection neurons undergo incomplete radial migration, resulting in the disruption of the cortical laminae, and leading to poor motor function, seizure and varying levels of intellectual disabilities (Kato and Dobyns, 2003; Moffat et al., 2015). However, the underlying mechanisms by which abnormal neuronal migration and positioning generate defects in circuit organization and behavior are poorly understood.

The motor circuits generated by the vertebrate facial branchiomotor (FBM) neurons are an excellent model to investigate the mechanisms linking neuronal position to circuit function. In mouse and zebrafish, FBM (nVII) neurons undergo tangential migration from rhombomere 4 (r4) into r6 (mouse) and r6/7(zebrafish), with their axons exiting the hindbrain from r4 (Chandrasekhar et al., 1997; Chandrasekhar, 2004; Guthrie, 2007). The trigeminal (nV) motor axons (whose cell bodies are located in r2 and r3) and FBM motor axons innervate the jaw and gill muscles in zebrafish, and jaw muscles, facial muscles and tongue in mammals (Lumsden and Krumlauf, 1996; Higashijima et al., 2000; Chandrasekhar, 2004; Song, 2007).

Potential mechanisms linking aberrant neuronal position and defective circuit function have been examined in FBM migration mutants in mice (Thoby-Brisson et al., 2012) and zebrafish (McArthur and Fetcho, 2017). In *Looptail* (*Lp*) mutant mice, defective in function of the Wnt/Planar Cell Polarity (PCP) gene *Vangl2*, FBM neurons fail to migrate caudally out of r4 (Glasco et al., 2012). Since the embryonic parafacial nucleus (e-pF), a neuronal population that controls breathing, develops adjacent to the FBM neurons, mispositioning of the FBM neurons in *Lp* mutants could impact e-pF development and function. Even though e-pF neurons showed a broad aberrant distribution in *Lp* mutants, they developed characteristic network properties, and established functional connections with other brainstem oscillators regulating breathing (Thoby-Brisson et al., 2012). This work suggests that functional outputs of neuronal circuits critical for survival (such as breathing) are unlikely to be severely impacted by defective neuronal positioning.

In a similar study done with zebrafish, McArthur and Fetcho (2017) investigated the developmental and functional consequences of failed FBM neuron migration using mutants defective in Wnt/PCP components (Gray et al., 2011). In *landlocked* (*llk*) and *prickle1b* (*pk1b*) mutants, carrying loss of function mutations in Scribble1a and Prickle1b, respectively, FBM neurons failed to migrate out of r4, yet appear to innervate jaw muscles normally (Wada et al., 2005; Mapp et al., 2010; McArthur and Fetcho, 2017). Importantly, although FBM cell bodies and dendrites were mispositioned in the mutants, the organization of neurons within the motor nucleus according to age and target muscle was not affected. Remarkably, electrophysiological recording and Ca^2+^ imaging of individual FBM neurons and groups of FBM neurons, respectively, showed that activity patterns were similar between mutants and their wildtype siblings. Moreover, opercular movement, generated by FBM-innervated muscles, was essentially normal in mutants. This study again suggests that the functional output of neuronal networks regulating critical functions is resilient to changes in neuronal position and highlights the robustness of neuronal networks mediating essential physiological functions. We have tested this idea of resiliency again in our studies and report that mispositioning of FBM neurons is associated with pronounced changes in the branchiomotor circuit and its output.

To examine the functional consequences of defective FBM migration, we used the zebrafish mutant *off-limits* (*olt*), which inactivates *frizzled3a* (*fzd3a*), a Wnt/PCP gene (Wada et al., 2006). In *olt* mutants, FBM neurons fail to migrate caudally out of r4. Genetic mosaic analysis showed that *fzd3a* functions in a non-cell autonomous manner during FBM migration; consistent with this, *fzd3a* is expressed broadly in the hindbrain and surrounding tissues during the migratory period (Wada et al., 2006). Interestingly, *olt* mutants look morphologically normal and the homozygous mutant larvae can grow into viable and fertile adults (Wada et al., 2006), suggesting that the consequences of defective FBM positioning (and of *fzd3a* loss of function) on survival are minimal. Nevertheless, it is important to evaluate the effects of FBM mispositioning on branchiomotor circuit organization, assembly and output.

We evaluated the functional output of the FBM neurons using a jaw movement assay developed in our lab. *Off-limit* mutants exhibited reduced gape (opening and closing the mouth) frequency compared to their wildtype siblings. This decrease in gape frequency correlated with reduced food intake; i.e., *olt* mutants ate poorly. While patterns of innervation and neuromuscular junction (NMJ) structure were largely similar between *olt* mutants and wildtype siblings, there were subtle defects in axon fasciculation and neuronal activity. These studies establish the foundation for further investigating the role of neuronal position on circuit function.

## Materials and Methods

### Animals

Adult zebrafish (*Danio rerio*) were maintained according to standard protocols (Westerfield, 1995) approved by the Animal Care and Use Committee at the University of Missouri. Embryos were obtained through natural mating and were incubated in E3 medium at 28.5°C. Embryos were staged by counting somites at 16–18 hpf (hours post fertilization) (Kimmel et al., 1995), and pigmentation was prevented by 0.003% phenylthiourea treatment from 18 hpf.

The *Tg*(*isl1:GFP*) line (Higashijima et al., 2000) expresses GFP in all branchiomotor neurons (nV, nVII, nIX, and nX) throughout development, and was used to characterize the developmental acquisition of jaw movement. *Tg*(*zCREST1:mRFP*) expresses membrane RFP in branchiomotor neuron cell bodies and axons (Mapp et al., 2010). *Tg*(α*-actin:GFP*) expresses GFP in most muscles, including the jaw, gill and ocular muscles (Higashijima et al., 1997). *Tg(zCREST1:GCaMP6s)* was generated by Tol2 transgenesis. The Tol2 destination vector was generated by Gateway Cloning of p5E-zCREST1, pENTR-GCaMP6s, and p3E-polyA constructs. This line expresses GCaMP6s in branchiomotor neurons at low levels until 48 hpf, and at a much higher level between 4–7 dpf (days post fertilization). The *off-limits* (*olt^*rw*689^*) mutant allele contains a loss-of-function missense mutation in the *frizzled3a* (*fzd3a*) gene (Wada et al., 2006).

### Jaw Movement Assay

After embryos hatched at 2 dpf, they were screened based on facial branchiomotor (FBM) neuron migration. Larvae at 2 dpf were placed in 28.5°C 14-h light and 10-h dark cycles (LD 14:10) incubator. Larvae at 3, 5, 7, or 9 dpf were placed into a multi-lane 1.2% agarose mold petri dish minimizing liquid transfer. Low melting agarose (2%) in E3 was applied gently over the larvae, which were oriented laterally into the agarose troughs. 1.2% agarose in E3 [Agarose LE (Gold Biotechnology)] was applied over the lower trunk region to trap the larvae. Excess agarose was removed from the head region, allowing for full movement of the lower jaw (Supplementary Movies 1–5). Each larva was recorded under brightfield with an Olympus SZX12 stereomicroscope (90× magnification) fitted with a Retiga 2000 camera (QImaging, Inc.), for 1 min after jaw movement was observed (18 frames per second, 600 frames) and removed after 10 min if no jaw movement occurred. For experiments involving *olt* mutants, the person imaging the larvae was blinded to the larvae’s genotypes, ensuring that each movie was analyzed in an unbiased manner. Jaw movement was detected using custom software (Supplementary Figure 1) (Kassim et al., 2018). Gape frequency, a measure of the rate of mouth opening due to the deflection of the lower jaw, was calculated by the software by dividing the total number of detected jaw deflections by the duration (in seconds) of the recording.

### Food Intake (Feeding) Assay

Our procedure is extensively described in Allen et al. (2017). Briefly, 10–15 larvae were placed in 5 ml of E3 medium containing 2 mg Larval fish food (Zeigler <100 microns) coated in yellow fluorescent microspheres (Molecular Probes/Thermo Fisher) for 3 h. At the end of the feeding period, larvae were anesthetized with 0.02% tricaine (Western Chemical Inc.), mounted laterally, and scored based on the amount of fluorescent food in the gut. Larvae were examined with an Olympus SZX12 stereomicroscope equipped with epifluorescence optics at 40–90× fitted with a Retiga 2000 camera (QImaging, Inc.) and assigned a score ranging from 0–3. A feeding score of ‘0’ corresponded to the larvae with no fluorescent signal within the gut, while a score of ‘3’ corresponded to fluorescence throughout the gut (Figure 4A). Two individuals independently scoring each larva were blinded to the treatment and genotype.

### Swimming Assay

At 2 dpf, mutant larvae and wildtype siblings were separated based on FBM migration phenotype and incubated at 28.5°C. At 6 dpf, larvae were placed in individual wells of a 24-well plate and observed at room temperature (21–22°C). The DanioVision system and EthoVision XT 8.0 locomotion tracking software (Noldus) were used to measure the distance moved and moving duration. Larvae were allowed to acclimate to the dark for 30 min, and distance moved and moving duration parameters were analyzed (Figures 4G,H).

### Alcian Blue Staining

The alcian blue staining method (Nissen et al., 2006) was adapted to visualize the cartilage structure in the larval head. Larvae were fixed in 4% paraformaldehyde (PFA) overnight at 4°C, washed twice with PBST (PBS containing 0.1% Tween20) and dehydrated through 50% methanol (MeOH) in PBST into 100% MeOH. This was followed by overnight staining in 0.15% alcian blue (Sigma) in ethanol (EtOH) solution at room temperature with gentle agitation. Larvae were washed with 100% EtOH, rehydrated through 50% EtOH/PBST into 100% PBS and digested in 0.05% trypsin (in 1 × PBS) for 24 h, and rinsed in PBST. Larvae were bleached in 3% H_2_O_2_/1% KOH at room temperature, checking every 5 min until desired transparency was reached and transferred to 70% glycerol through a glycerol series. Larvae were imaged on a Leica DM widefield microscope. Jaw cartilages were analyzed for length and angles related to the head skeleton anatomy with ImageJ (NIH).

### Immunostaining and Analysis of Neuromuscular Junctions

Our procedure was adapted from Hunter et al. (2011). Zebrafish expressing GFP in the jaw muscles [*Tg*(α*-actin:GFP*)] were used in the analysis. The pre-synaptic compartment of NMJs was stained using a monoclonal antibody against the pre-synaptic protein SV2a (Developmental Studies Hybridoma Bank, 1:200 dilution) and a goat anti-mouse Alexa Flour 568 (Life Technologies, 1:500 dilution) as the secondary antibody. The jaw muscles were stained using rabbit anti-GFP (Invitrogen, 1:2000 dilution) as the primary antibody, and chicken anti-rabbit Alexa Fluor 488 (Invitrogen; 1:500 dilution) as the secondary antibody.

Post-synaptic structures at NMJs were visualized using a protocol provided by Dr. Diane Sepich (Washington University School of Medicine, St. Louis), modified from a previous study (Panzer et al., 2005). Transgenic larvae expressing GFP in the jaw muscles were used in these studies. Live larvae were incubated in Alexa Fluor 594-conjugated α-bungarotoxin (αBTX; 10 μg/ml; Invitrogen) for 30 min in E3 medium containing 15% DMSO.

Larvae were embedded in 1% LMP agarose (SV2) or anesthetized in 0.02% Tricaine (αBTX) for imaging. Bidirectional Z-stack images (1 μm thickness, 140–200 slices) were acquired with a Leica TCP SP8 MP inverted confocal microscope with a 20×/0.75 water immersion objective. Confocal stacks were processed in two channels to obtain 3D images with Leica Application Suite X (LAS X). 3D images from the GFP channel (468 nm) (jaw muscles) were used as a mask overlay to examine the 3D images of the SV2-labeled presynaptic and αBTX-labeled postsynaptic structures in the 568/594 nm channel, and analyzed using LAS X (Supplementary Figure 3).

### Axon Imaging and Analysis

Double transgenic zebrafish expressing membrane RFP [*Tg*(*zCREST1:mRFP*)] in the branchiomotor axons and GFP in the jaw muscles [*Tg(*α*-actin:GFP)*], were used to analyze axon morphology. Larvae were anesthetized with 0.02% tricaine, and bidirectional Z-stack images (1 μm thickness, 15–25 slices) were taken with a Leica TCP SP8 MP confocal microscope with a 20×/0.75 water immersion objective. Leica Application Suite X (LAS X) software was used to generate 3D images from the stacks of confocal images from the RFP channel (axons) and GFP channel (jaw muscles). Axon length and branching number in the lateral view were traced in the 3D image by examining each slice. Images were analyzed by LAS X and Fiji (NIH) (Supplementary Figure 5 and Supplementary Movie 6). In ventral views, the morphologies of axons extending on the ih muscle were used to define Normal Fasciculation (NF), when 1–2 thick axon fascicles were seen, and Defasciculation (DF), when >2 thinner axon fascicles were seen, sometimes extending outside the muscle (Figure 5). To measure the number and thickness of axon fascicles on the ih muscle, we used Fiji to “section” each ih muscle at 3 equidistant points perpendicular to its long axis and traced the intensities of the green (muscle) and red (axon) fluorescence along the width (short axis) of the muscle (Supplementary Figure 6). Because the shoulders of many peaks precluded measuring the width at the base of the peak (fascicle thickness), we measured the widths at half-maximal intensity, and added the widths per section to obtain total axon fascicle thickness. We obtained data from 6 sections per larva (3 per side), and 5 larvae each for wildtype and *olt* mutants. For WT, we used 3 larvae classified as Normal Fasciculation (NF) and 2 larvae classified as Defective Fasciculation (DF) (Figure 5). For *olt* mutants, we used 1 NF and 4 DF larvae.

### Calcium Imaging and Analysis

Double transgenic larvae expressing the calcium indicator GCaMP6s and membrane RFP in the branchiomotor neurons [*Tg*(*zCREST1:GCaMP6s*) generated in our lab] were used for this analysis. Larvae were maintained in an LD 14:10 incubator. One hour before imaging, larvae were injected with 4.6 ng of α-bungarotoxin (Alomone Labs) to paralyze and prevent twitching. Larvae were mounted dorsally on a coverglass-bottom petri-dish in 0.2% agarose in E3, and covered with E3 after setting. Imaging was performed using a Leica TCS SP8 MP inverted confocal microscope with a 20×/0.75 water immersion objective. Larvae were imaged at 2 frames per second for 5 min (Supplementary Movies 7, 8). Images were analyzed using Fiji (NIH), and regions-of-interest (ROIs) were manually drawn corresponding to FBM neurons and trigeminal neurons based on GCaMP expression and location in the hindbrain. Only recordings from larvae with blood flow after the imaging session were used for analysis.

GCaMP images were single plane confocal images. We chose a focal plane for GCaMP imaging based on the maximum number of mRFP-expressing FBM neurons that could be visualized under confocal imaging. Under these conditions at 7 dpf, the number of trigeminal (nV) motor neurons imaged in the same focal plane was variable due to differences in the dorso-ventral positions of the nV and nVII cell bodies and the positioning of the larva. To draw the ROIs, the recording was advanced to the first frame that showed strong GCaMP fluorescence. ROIs were manually drawn for a group of neurons. Separate ROIs were drawn for the two sides. The same ROIs were used for all frames of the recording. Since the GCaMP signals were synchronous between the two sides, the raw values in every frame for the neurons on the two sides were pooled for analysis. The same ROI mask was used for background subtraction by placing it in an FBM-adjacent region with no RFP-labeled motor neurons. A similar procedure was followed for generating the ROI and for background subtraction for trigeminal motor neurons. Background subtraction was performed on every frame of the recording, yielding the background-corrected trace. Because several of the peaks seen in these traces were broad, precluding an automatic counting of the Ca^2+^ events, they were counted manually by two independent observers watching the time-lapse recordings. For the subset of FBM recordings that were also used for trigeminal neuron analysis (Figure 7), we evaluated their signal to noise characteristics as described (McArthur and Fetcho, 2017). In brief, an average background fluorescence value (*F*_b_) for each recording was generated by segmenting the movie into four equal durations, calculating an average background value for each segment, and averaging the four segment means. The background-corrected fluorescence value (*F*) for each frame was used to calculate (Δ*F* = *F* − *F*_b_)/*F*, which revealed that 60–70% of the total fluorescence represented signal from the motor neurons (Supplementary Figure 7).

### Statistical Analysis

**FIGURE 1:**
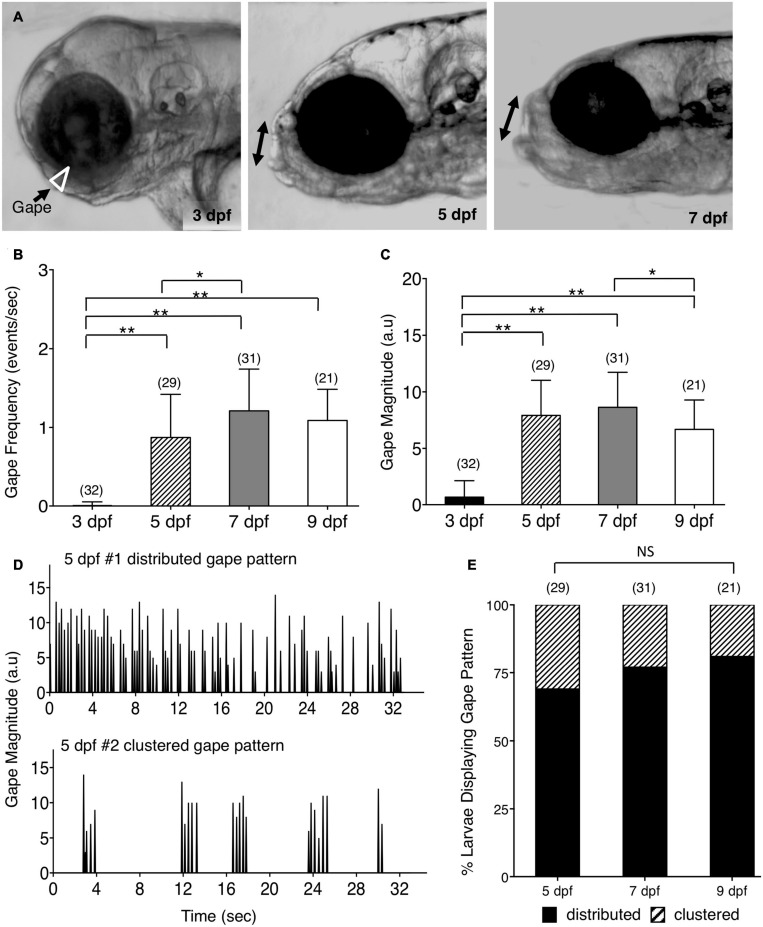
Ontogeny of lower jaw movement in wildtype zebrafish larvae. **(A)** *Tg*(*isl1:GFP*) larvae from 3, 5, and 7 dpf were mounted in a lateral position, under a brightfield microscope, in agarose with the head free to move, and the opening of the mouth due to lower jaw movement (gape; white triangle and double arrows) was recorded and analyzed using custom software. **(B,C,E)** Pooled data from 3 experiments (number of larvae in parenthesis). **(B)** Gape frequency, increased sharply from 3 to 7 dpf, plateauing by 9 dpf. **(C)** Gape magnitude, a measure of the amount of jaw movement, increased sharply between 3 and 5 dpf, and plateaued thereafter. **(D)** There were two patterns of gape events – Distributed, with jaw movements distributed uniformly throughout the observation period, and Clustered, with tightly clustered jaw movements with intervals of inactivity. **(E)** The distributed gape pattern was predominant at every age examined, and there was no significant difference between 5, 7, and 9 dpf. Statistical analysis was carried out with Chi-square test and One-way analysis of variance (ANOVA) with *post hoc* Tukey’s HSD (honest significance difference). NS, not significant, *p < 0.05, **p < 0.01.

For jaw movement analysis from 3 to 9 dpf (Figure 1), Chi-square test and one-way analysis of variance (ANOVA) with *post hoc* Tukey’s HSD (honest significance difference) were employed. For comparisons between *olt* mutants and wildtype siblings (various Figures), two-tailed student *t*-test or Chi-square test (Figure 3) or Fisher’s exact *t*-test (Figure 5) was used. See Figure legends for details.

## Results

### Ontogeny of Lower Jaw Movement in Wildtype Zebrafish Larvae

The cranial motor neurons located in the vertebrate hindbrain, called branchiomotor neurons, innervate muscles of the jaw, face, and neck (Chandrasekhar, 2004; Guthrie, 2007). In zebrafish, they innervate muscles of the lower jaw and gills. To investigate the consequences of defective positioning of facial branchiomotor (FBM) neurons on circuit function and behavioral output, we developed a quantitative assay to evaluate jaw movement in zebrafish larvae. The trunks of 3–9 days post fertilization (dpf) larvae were mounted in agarose in a lateral orientation with the head free to move (Figure 1A). The spontaneous opening of the mouth due to lower jaw movement, termed gape, was recorded by time-lapse imaging and analyzed using custom gape detection software (Kassim et al., 2018) (Supplementary Figure 1 and Supplementary Movies 1–3). In 3 dpf *Tg(isl1:GFP)* (Higashijima et al., 2000) larvae, there was no jaw movement. Gape frequency, the rate of mouth opening, increased sharply between 3 and 5 dpf, increased further by 7 dpf, and maintained a stable high level up to 9 dpf (Figure 1B). Gape magnitude, a measure of the mouth opening, also increased sharply between 3 and 5 dpf and stabilized thereafter (Figure 1C). Interestingly, the gape frequency in 5–9 dpf larvae exhibited a broad range due to the presence of two distinct jaw movement patterns: a distributed pattern with gape events uniformly distributed throughout the observation period, and a clustered pattern with tightly clustered events (<10) with intervals of inactivity (Figure 1D). The proportions of the two patterns were similar between different ages, with the distributed pattern being the predominant one (Figure 1E). The ratio of eye diameter to head height at the jaw level did not change significantly between 3 and 9 dpf (Supplementary Figure 2; data not shown), indicating that the changes in gape magnitude are not due to differential growth of the jaw with age. These data demonstrate that the emergence and establishment of jaw movement between 3–9 dpf is a developmentally reproducible behavioral output of the branchiomotor neuron circuits.

### Changes in Branching and Pre-synaptic Structures of Branchiomotor Axons May Drive Increases in Jaw Motor Output

The developmentally regulated changes in jaw gape frequency and gape magnitude may reflect the progression of specific events associated with the organization of the branchiomotor circuits and their innervation of the jaw muscles. While trigeminal branchiomotor (nV) and FBM (nVII) axons have fully extended over the mandibular and hyoid muscles by 3 dpf (Higashijima et al., 2000), long before overt jaw movement, further expansion of outgrowth patterns after 3 dpf, and pre-and post-synaptic changes at the neuromuscular junctions (NMJs) between 3 and 9 dpf have not been studied.

**FIGURE 2:**
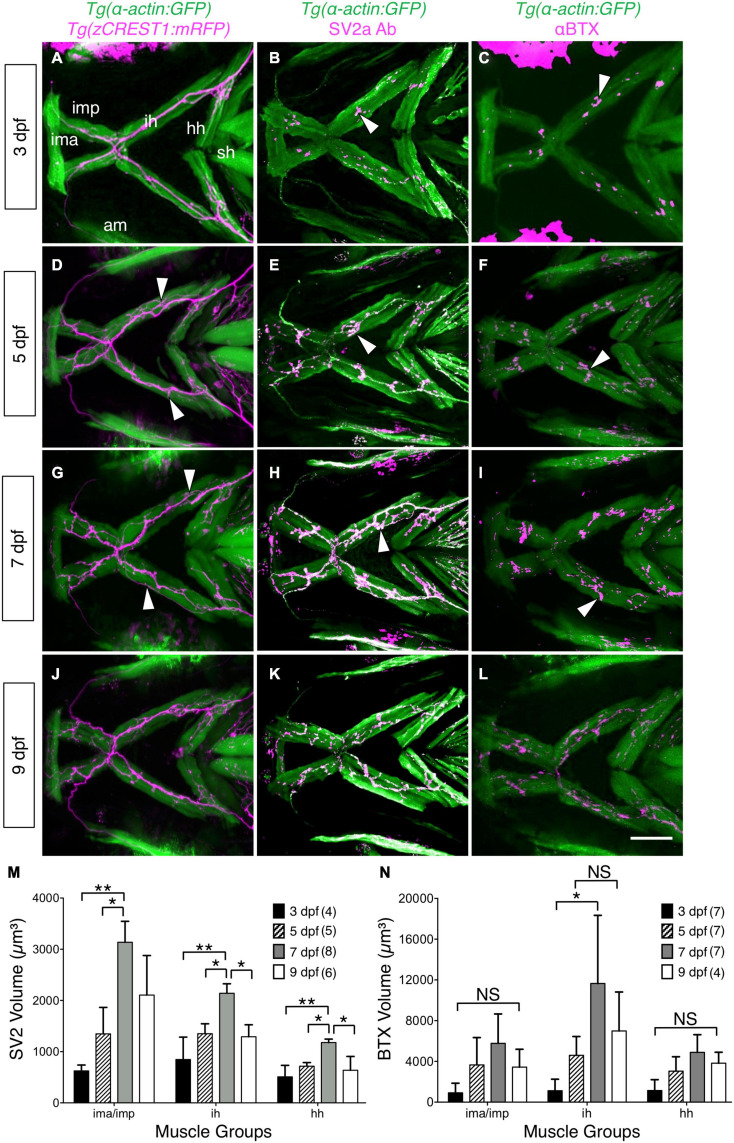
Developmental changes in branchiomotor axon branching and synaptic structures at the jaw neuromuscular junctions. Ventral views with anterior to the left of the jaw musculature. **(A,D,G,J)** Axon (magenta) outgrowth on jaw muscles (green) in 3, 5, 7, and 9 dpf *Tg* (α*-actin:GFP*); *Tg* (*zCREST1:mRFP*) larvae. 3D rendering of muscles and axons were overlaid to determine axon position relative to the muscles. Fine axon branches (white arrowheads) across the jaw muscles increased as larvae aged. **(B,E,H,K)** SV2a antibody (magenta) labeled presynaptic structures on the jaw muscles (green) in 3, 5, 7, and 9 dpf *Tg*(*isl1:GFP*)*; Tg*(α*-actin:GFP*) larvae. 3D rendering was used to visualize the presynaptic regions in contact with the muscles (arrowheads), and calculate their volumes. **(M)** Presynaptic volumes increased greatly from 3 to 7 dpf larvae, especially for ima/imp and ih muscles. There was a significant increase from 5 to 7 dpf in the ima/imp, ih, and hh muscles. Both ih and hh muscles had a significant decrease in presynaptic volume from 7 to 9 dpf larvae. **(C,F,I,L)** Ventral view with anterior to the left. Acetylcholine receptor (AChR) clusters (arrowheads) were labeled with alpha-bungarotoxin (αBTX) (magenta) on the jaw muscles (green) in live 3, 5, 7, and 9 dpf *Tg*(α*-actin:GFP*) larvae. **(N)** Although AChR cluster volumes on all muscles tend to increase from 3 to 7 dpf, these changes were not significant. Data pooled from 3 to 5 experiments (number of larvae in parenthesis). Statistical analysis was carried out with Chi-square test and One-way analysis of variance (ANOVA) with *post hoc* Tukey’s HSD (honest significance difference). NS, not significant; *p < 0.05, **p < 0.01. ima/imp, intermandibularis anterior/intermandibularis posterior; ih, interhyal; hh, hyohal; sh, sternohyoideus.

We examined whether branchiomotor axon outgrowth patterns ramified further between 3 and 9 dpf, when jaw movements become fully established. We imaged these structures in live, embedded *Tg* (α*-actin:GFP*); *Tg* (*zCREST1:mRFP*) larvae containing GFP-expressing muscles (Higashijima et al., 1997) and mRFP-expressing branchiomotor axons (Mapp et al., 2010) (see section “Axon Imaging and Analysis”; Supplementary Movie 6). While the branchiomotor axon fascicles extending on the intermandibularis posterior (imp), interhyal (ih), and hyohal (hh) muscles were largely similar in 3–9 dpf larvae (Figures 2A,D,G,J), there was an increase in branching of the fascicles between 3 and 5 dpf (5 larvae per time point), coinciding with the increase in gape frequency and magnitude. A prominent FBM axon branch innervating the superficial membranous muscles (Higashijima et al., 2000) also emerged between 3 and 5 dpf (Figures 2A,D).

While FBM axon branching increases between 3 and 5 dpf, the increase in gape frequency and gape magnitude during this period could also result from changes at the NMJs on the imp, hh, and ih muscles. Therefore, we examined presynaptic structures at the jaw muscles by staining *Tg* (*isl1:GFP*); *Tg* (α*-actin:GFP*) larvae with an antibody against Synaptic Vesicle protein 2a (SV2a Ab) (Portela-Gomes et al., 2000), and quantified their volumes using Leica LAS X software (see section “Immunostaining and Analysis of Neuromuscular Junctions”; Supplementary Figure 3). There was a significant increase in presynaptic volumes on ima/imp, hh and ih muscles from 3 to 7 dpf (Figures 2B,E,H,M), concomitant with the increases in gape frequency and magnitude (Figures 1B,C). Interestingly, there was significant decrease in presynaptic volumes on these muscles from 7 to 9 dpf (Figures 2H,K,M), corresponding to a decrease in gape magnitude by 9 dpf (Figures 1B,C).

Acetylcholine receptors (AChR) in the postsynaptic structures at the jaw NMJs were stained in live larvae with alpha-bungarotoxin (αBTX) conjugated with Alexa Fluor 568 (Panzer et al., 2005). Although there was high variability, the volumes of AChR clusters on the imp, hh, and ih muscles exhibited large increases from 3 to 7 dpf (Figures 2C,F,I,N) and a subsequent decrease by 9 dpf.

Taken together, these results suggest that increases in FBM axon branching on the jaw muscles and a significant increase in presynaptic volumes at the jaw NMJs can contribute to the onset of sustained jaw movement by 5 dpf.

### Jaw Movement Is Defective in the *off-limits* (*olt*) Neuronal Migration Mutant

**FIGURE 3:**
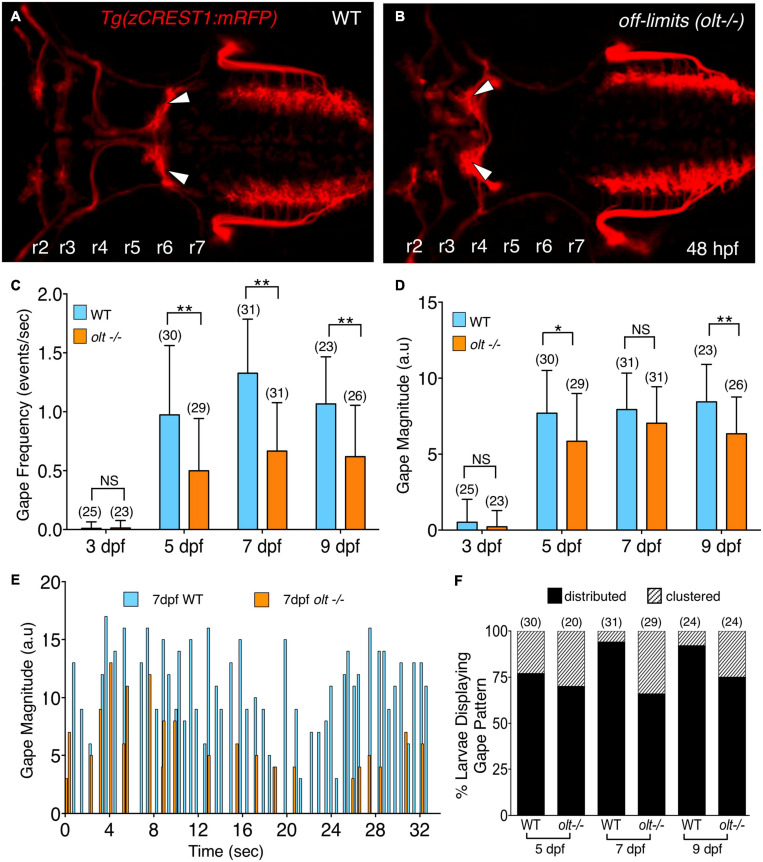
*olt* mutants have reduced jaw movement. **(A,B)** Dorsal views with anterior to the left of the hindbrain of 48 hpf *Tg(zCREST1:mRFP)* in the *olt* background. **(A)** Wildtype (WT) sibling with facial branchiomotor (FBM) neurons (arrowheads) migrating into rhombomere 6 (r6). **(B)** In the *olt* mutant, FBM neurons (arrowheads) fail to migrate out of r4. **(C)** Gape frequencies in 5, 7, and 9 dpf *olt* mutant larvae were significantly reduced compared to wildtype siblings. Notably, the plateauing of gape frequencies after 7 dpf occurred normally in *olt* mutants. **(D)** Gape magnitude was significantly reduced in *olt* mutants compared to wildtype siblings at 5 and 9 dpf, but not at 7 dpf. **(E)** Gape events in representative 7 dpf *olt* mutant and wildtype sibling larvae showing reduced gape frequency in the mutant. **(F)** Gape event patterns were similarly proportioned between wildtype and *olt* mutant larvae at 5 dpf, with the distributed pattern being the predominant one. In 7 and 9 dpf wildtype larvae, the distributed pattern was almost exclusively seen. Statistical analysis was performed with a two-tailed student *t*-test **(C,D)** or Chi-square test **(F)**. NS, not significant, *p < 0.02, **p < 0.001. Data pooled from 9 experiments (number of larvae in parenthesis).

A reduction in the number or loss of branchiomotor neurons, including FBM neurons, can severely disrupt food intake (Allen et al., 2017; Kassim et al., 2018). However, mispositioning of FBM neurons in *landlocked* mutants did not affect opercular muscle movement (McArthur and Fetcho, 2017). To further investigate a role for FBM neurons in generating branchiomotor circuit output, we tested whether jaw movement was affected in *off-limits* (*olt*) mutants, which are deficient in *frizzled3a* (*fzd3a*) function, and where FBM neurons fail to migrate caudally out of r4 (Figures 3A,B) (Wada et al., 2006). Jaw movement was imaged and analyzed in wildtype siblings and *olt* mutant larvae as described earlier. There was a significant decrease in gape frequency in 5, 7, and 9 dpf *olt* mutants, with wildtype siblings exhibiting gape frequencies roughly double that of *olt* mutants at every age (Figure 3C and Supplementary Movies 4, 5). While gape frequencies in *olt* larvae were significantly lower than wildtype at all ages, the sharp increase in gape frequency between 3 and 5 dpf, and the plateauing of the frequencies between 7 and 9 dpf occurred normally in mutants (Figure 3C). The effects on gape magnitude were mixed, with significant reduction in *olt* mutants at 5 and 9 dpf, but not at 7 dpf (Figure 3D). Furthermore, the proportions of “distributed” and “clustered” patterns of gape events were similar between wildtype and *olt* mutant larvae at all ages, although the “distributed” pattern was overrepresented in 7 and 9 dpf wildtype larvae (Figure 3E). Importantly, gape frequency was significantly lower in *olt* mutants even when the “distributed” and “clustered” patterns were considered separately (Supplementary Figure 4), indicating that the reduced gape frequency in mutants is not an artifact of the overrepresentation of the “distributed” pattern in wildtype larvae. These results suggest that while the proper migration and positioning of FBM neurons are important factors in generating jaw movement at a normal rate, they appear to play a minor or no role in the developmental onset of jaw movement or its magnitude, especially at 7 dpf when larvae are actively foraging.

### Defective Jaw Movements in *olt* Mutants Are Associated With Reduced Food Intake

**FIGURE 4:**
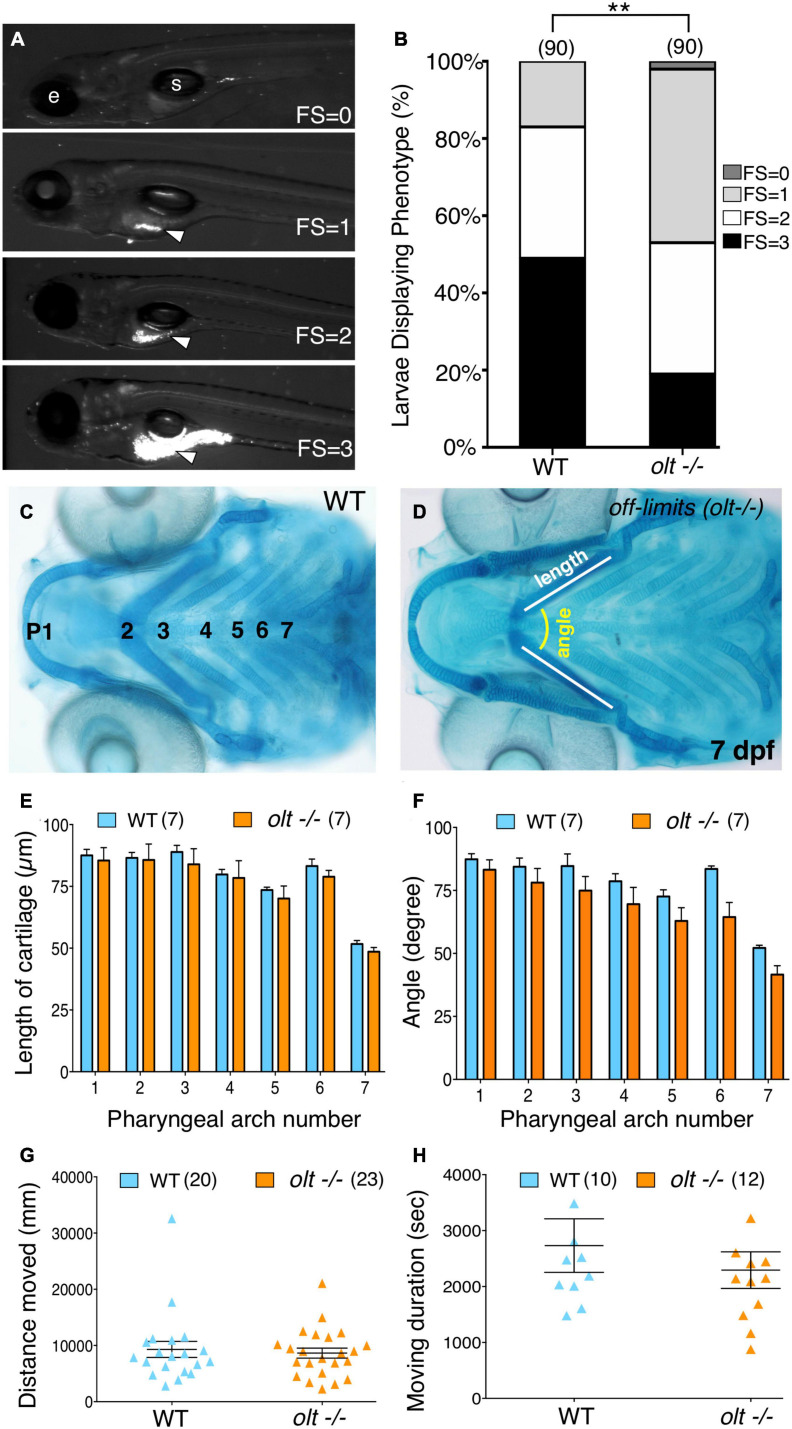
Defective jaw movements greatly reduce food intake in *olt* mutants. **(A)** A semi-quantitative food intake assay for zebrafish larvae. Lateral views of 7 dpf larvae fed yellow–green fluorescent microspheres coated with fish food for 3 h. The fluorescent contents (arrowheads) in their guts were visualized under GFP epifluorescence, and ranged from no food with a feeding score (FS) of 0, less than 25% of the gut FS = 1, 50% of the gut (FS = 2), to a full gut (FS = 3). e, eye; s, swim bladder. **(B)** Distribution of feeding scores showing that a population of *olt* mutants ate significantly less than wildtype siblings. Pooled data from 3 experiments. Chi-square test used for testing significance (**p < 0.01). **(C,D)** Cartilage morphology at 7 dpf showing that the various elements develop and pattern normally in *olt* mutants. **(E,F)** Pooled data from 4 experiments. The pharyngeal arch (numbered) was measured for **(E)** length (white line in **D**) and **(F)** angle (yellow arc in **D**) in wildtype siblings and *olt* mutants. There was no difference in cartilage elements’ lengths or the angles subtended by the elements between *olt* mutants and wildtype siblings. **(G,H)** Swimming parameters in 6 dpf larvae. **(G)** distanced moved and **(H)** moving duration were analyzed and compared between *olt* mutant and wildtype sibling larvae with the DanioVision and EthoVision locomotion tracking software (Noldus). There was no significant difference in swimming distance or duration between *olt* mutants and wildtype siblings. Pooled data from 2 experiments. Number of larvae in parenthesis. The two-tailed student *t*-test was performed to test for significance **(E–H)**.

Given the reduced jaw movement in *olt* mutant larvae, we wondered whether their ability to track and capture food particles (food intake) was also reduced. We employed a robust and sensitive feeding assay developed in our lab (Allen et al., 2017) to visualize fluorescent microsphere-coated food particles within the gut of immobilized larvae. Actively swimming 7 dpf larvae were fed fluorescent food for 3 h and assigned a feeding score (FS) from 0 to 3, representing the distribution of the fluorescent microspheres within the gut, ranging from no fluorescence (FS = 0) to a fully fluorescent gut (FS = 3) [Figure 4A; see section “Food Intake (Feeding) Assay”]. Food intake in the *olt* mutant population was significantly reduced compared to wildtype (Figure 4B). While some *olt* mutant larvae ate just as well as most wildtype larvae, the distribution was greatly skewed toward low food intake (FS = 0 and 1) in mutants compared to wildtype siblings.

Given the broad expression pattern of *fzd3a* (Wada et al., 2006), poor food intake in *olt* mutants may be a pleiotropic effect of the mutation resulting from deformation of the jaw or an inability to swim normally. Alcian blue staining of jaw cartilage elements in 7 dpf *olt* mutant larvae and wildtype siblings revealed no differences in the lengths of various cartilage elements or in the angles subtended by the elements between the two populations (Figures 4C–F), indicating that the lower jaw developed and was patterned normally in mutants. Swimming activity of 6 dpf larvae imaged and analyzed with the DanioVision and EthoVision locomotion tracking software (Noldus; see section “Swimming Assay”) revealed no differences in swimming distance or swimming duration between *olt* mutants and wildtype siblings (Figures 4G,H). Taken together, these data suggest strongly that the reduced food intake seen in *olt* mutants results specifically from the reduced jaw movement of mutant larvae.

### FBM Axon Outgrowth to Jaw Muscles Occurs Normally in *olt* Mutants

**FIGURE 5:**
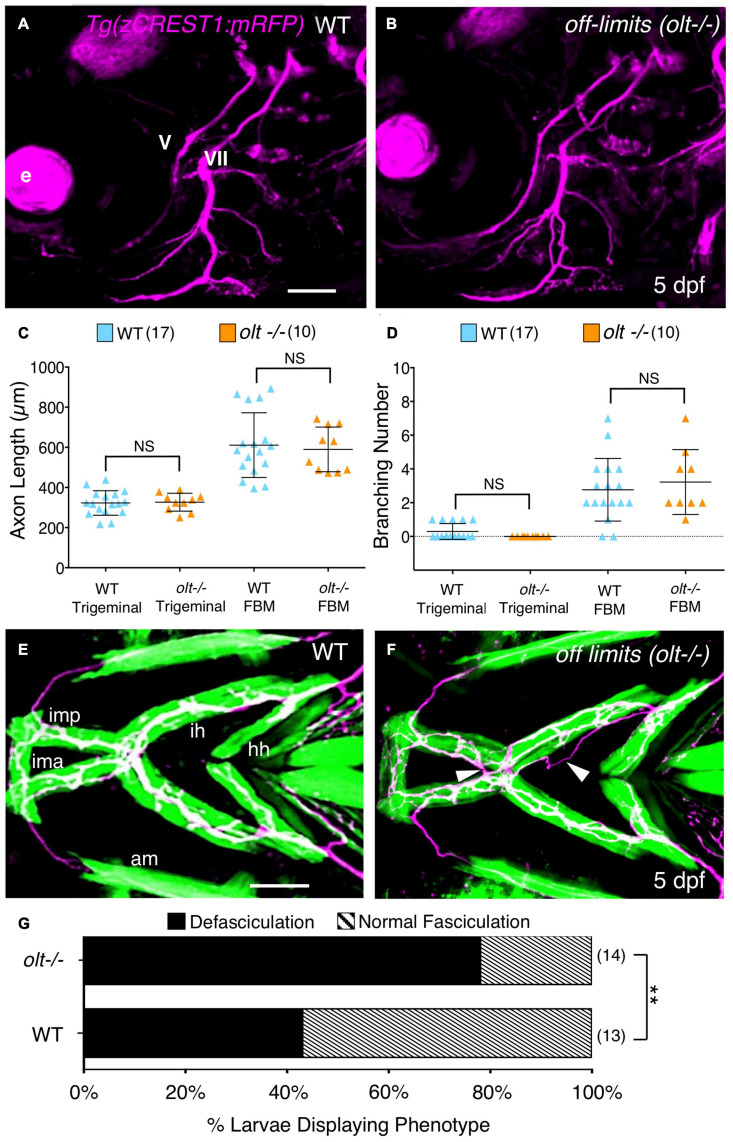
Axon guidance and outgrowth are unaffected in *olt* mutant larvae. **(A,B)** Lateral view with anterior to the left of the hindbrains of 5 dpf *Tg(zCREST1:mRFP)* larvae. The morphologies of trigeminal (V) and FBM (VII) axons were analyzed and number of branches were calculated with Leica Application Suite X (LAS X) software. e, eye. **(C)** The total lengths of trigeminal motor axons and FBM axons and their branches were similar between *olt* mutants and wildtype siblings. **(D)** Trigeminal motor and FBM axon branching numbers showed no significant differences between wildtype siblings and *olt* mutants. Statistical analysis was performed with a two-tailed student *t*-test **(C,D)**. NS, not significant. Pooled data from 4 experiments (number of larvae in parenthesis). **(E,F)** Ventral view with anterior to the left of the jaw musculature in 5 dpf *Tg(zCREST1:mRFP); Tg(α-actin:GFP)* larvae showing motor axons (magenta) and jaw and gill muscles (green). **(E)** Thick motor axon fascicles were seen on the ima/imp, ih, and hh muscles in wildtype siblings. This phenotype was defined as Normal Fasciculation (NF). **(F)** In *olt* mutants, these axon fascicles appeared thinner and were frequently defasciculated (arrowheads), especially on the ih muscle. This phenotype was defined as Defasciculation (DF). **(G)** There was a preponderance of the defasciculated axon (DF) phenotype in *olt* mutants, and the proportion of normal fasciculation (NF) to defasciculated (DF) axons was significantly different between wildtype siblings and *olt* mutants. Statistical analysis was performed using Fisher’s two-tailed exact test. Data pooled from 4 experiments (number of larvae in parenthesis). **p < 0.0001. ima/imp, intermandibularis anterior/intermandibularis posterior; ih, interhyal; hh, hyohal. Scale bar **(A,E)**, 100 μm.

With some non-specific bases for reduced jaw movement in *olt* mutants ruled out, we tested whether specific aspects of the FBM axon pathway innervating the jaw muscles may be affected in mutant larvae. Although mutant FBM neurons appear to extend motor axons normally (Wada et al., 2006), their precise outgrowth and NMJs at the jaw muscles were not examined. Therefore, we 3D-reconstructed and quantified FBM axon outgrowth and branching in live 5 dpf *Tg(zGREST:GFP) olt* mutants using Leica LAS X software (see section “Axon Imaging and Analysis”; Supplementary Figure 5 and Supplementary Movie 6). Since the migration defect is restricted to FBM neurons, trigeminal motor (nV) axon outgrowth was used as a control because these neurons are positioned normally in mutants (Figures 5A,B). The total lengths (of the primary fascicle and branches) for trigeminal motor and FBM axons from their hindbrain exit points up to the jaw muscles were unaffected in *olt* mutants and wildtype siblings (Figures 5C,D). Additionally, there were no significant differences between *olt* mutants and wildtype siblings in the number of branches arising from trigeminal motor and FBM axons in *olt* mutants and wildtype siblings (Figures 5C,D). Interestingly, while FBM axons exhibited more branching, and hence had higher total length, than trigeminal motor axons, there were no differences between *olt* mutants and wildtype siblings. These results demonstrate that FBM axon outgrowth and extension on jaw muscles occur normally in *olt* mutants and cannot contribute to defective jaw movements in mutant larvae.

### Motor Axons Extending on the ih Muscle Are Frequently Defasciculated in *olt* Mutants

While branchiomotor axons extended normally to the jaw muscles in *olt* mutants, one cannot rule out subtle axonal defects at the muscles where the NMJs develop. Therefore, we used *Tg(zCREST:mRFP); Tg(α-actin:GFP)* fish to examine axonal outgrowth in 3D on the jaw muscles in 5 dpf larvae using Leica LAS X software. Axon branching on specific jaw muscles was isolated in 3D and viewed using a reference mask (see section “Axon Imaging and Analysis”; Supplementary Figure 3 and Supplementary Movie 6). We observed that motor axons, especially on the ih muscle, were organized into thicker fascicles in wildtype, whereas mutant axon fascicles were more numerous and thinner (Figures 5E,F and Supplementary Figure 6). Importantly, defasciculated axons, not situated on the muscle, were found at a significantly higher rate for *olt* mutant axons extending on the ih muscle (∼80%) than for wildtype axons (∼40%) (Figures 5E–G). These fasciculation defects have the potential to impact ih muscle activity and could contribute to jaw movement defects in *olt* mutants.

### NMJs on Jaw Muscles Appear to Be Unaffected in *olt* Mutants

**FIGURE 6:**
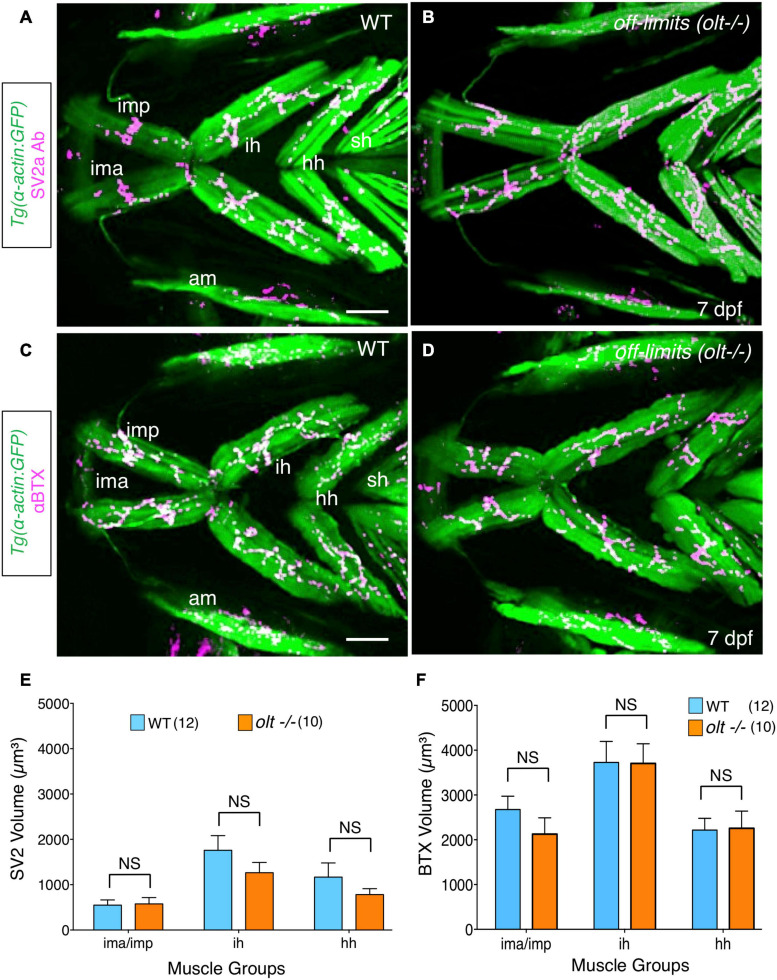
Neuromuscular junctions on jaw muscles in *olt* mutants are unaffected. **(A–D)** Ventral view with anterior to the left of the jaw musculature in 7 dpf *Tg(isl1:GFP);Tg(α-actin:GFP)* larvae. **(A,B)** SV2a antibody staining (magenta) of presynaptic structures on the jaw muscles (green). **(C,D)** Postsynaptic Acetylcholine receptor (AChR) clusters were labeled with alpha-bungarotoxin (αBTX) (magenta) on the jaw muscles (green) in live larvae. Volumes of the presynaptic structures and postsynaptic clusters were calculated from 3D renderings by using the muscle as a mask. There was no significant difference in the volumes of SV2a-stained structures **(E)** or αBTX-labeled structures **(F)** on any muscle groups between wildtype siblings and *olt* mutants. Data pooled from 3 experiments (number of larvae in parenthesis). The two-tailed student *t*-test was performed to test for significance. NS, not significant. ima/imp, intermandibularis anterior/intermandibularis posterior; ih, interhyal; hh, hyohal; am, adductor mandibularis; sh, sternohyoideus. Scale bar **(A,C)**, 100 μm.

Since motor axon fasciculation defects are evident at the ih muscle in *olt* mutants, it is possible that jaw NMJs are also affected in mutants. Furthermore, given that increased presynaptic volume may drive the developmentally regulated increase in gape frequency (Figure 2), reduced gape frequencies in *olt* mutants may result from smaller presynaptic structures. Therefore, we quantified presynaptic volumes (SV2a antibody staining) and postsynaptic acetylcholine clusters (αBTX labeling) in 7 dpf *olt* mutants as described earlier (Figure 2). The volumes of presynaptic structures on various jaw muscles were similar between *olt* mutants and wildtype siblings (Figures 6A,B). While there was a slight but consistent reduction in presynaptic volumes on the ih and hh muscles in mutants, these changes were not statistically significant (Figure 6E). Volumes of postsynaptic AChR clusters were largely unaffected on all muscle groups between 7 dpf *olt* mutants and wildtype siblings, and were essentially identical for the ih and hh muscles (Figures 6C,D,F). These results indicate that the reduced jaw movement of *olt* mutants does not result from deficits in NMJ structures formed by branchiomotor axons on jaw muscles.

### FBM Neurons Are Less Active in *olt* Mutants

**FIGURE 7:**
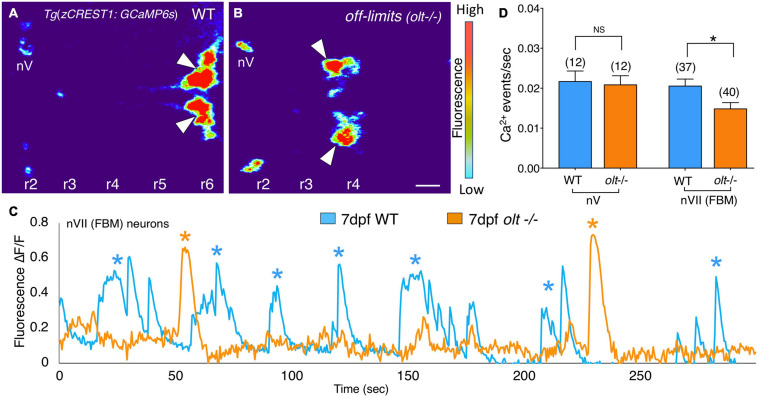
Facial branchiomotor neurons are less active in *olt* mutants. **(A,B)** Dorsal views, with anterior to the left, of the hindbrains of 7 dpf *Tg*(*zCREST1:GCaMP6s*)*;TgzCREST1:mRFP* larvae showing GCaMP fluorescence intensities. **(A)** In a wildtype larva, fluorescence can be seen in the FBM neurons (arrowheads) in r6, as well as the trigeminal motor neurons (nV) in r2. **(B)** In an *olt* mutant, fluorescence is evident in the nV neurons in r2 and in the FBM neurons (arrowheads) that have failed to migrate out of r4. **(C)** Overlay of GCaMP6s Δ*F*/*F* traces for a representative wildtype sibling (blue) and an *olt* mutant (orange) showing fewer Ca^2+^ events (asterisks) in the mutant. Amplitudes of the Ca^2+^ events were not affected. **(D)** The frequency of Ca^2+^ events in FBM neurons was significantly lower in *olt* mutants compared to wildtype siblings. The Ca^2+^ event frequency for trigeminal motor (nV) neurons was not affected in mutants. Data pooled from 6 experiments (number of larvae in parenthesis). The two-tailed student *t*-test was performed to test for significance, *p < 0.01.

Since extensive characterization of motor axon projections to, and NMJs on, jaw muscles revealed only subtle structural defects in *olt* mutants (Figure 5), we wondered whether FBM neuronal activity might itself be affected in mutants. To test this possibility, we introduced the *olt* mutant allele into the *Tg*(*zCREST1:GCaMP6s*) background where branchiomotor neurons express the calcium indicator GCaMP6s (see section “Animals”). The fish also expressed the *zCREST1:mRFP* transgene (Mapp et al., 2010). Wildtype and *olt* mutant larvae at 7 dpf were paralyzed by αBTX injection and mRFP-expressing FBM neuron clusters were identified in r6 (normal migration; wildtype) and r4 (defective migration; *olt* mutants), respectively. The neurons were imaged by time-lapse confocal microscopy (Figures 7A,B and Supplementary Movies 7, 8), and their activities were analyzed using Fiji (see section “Calcium Imaging and Analysis”). While the signal to noise ratios in these recordings were sufficient to reveal peaks of calcium-mediated fluorescence increase, calcium events were scored manually due to the broad peaks recorded by GCaMP6s fluorescence (Supplementary Figure 7). Periodic calcium events (asterisks) were seen throughout the observation period in both wildtype and mutant larvae; however, the events occurred less frequently in mutants (Figure 7C). The frequency of Ca^2+^ events was significantly lower in *olt* mutants compared to their wildtype siblings (Figure 7D and Supplementary Figure 7C). Importantly, the frequency of calcium events for trigeminal motor (nV) neurons located in r2 was similar between wildtype and *olt* mutants (Figure 7D), indicating that the reduction for FBM neurons is not a pleiotropic effect of the mutation. These results suggest reduced activity of FBM neurons may contribute to the jaw movement defect (lower gape frequency) of *olt* mutants.

## Discussion

Precise migration of newborn neurons is an integral part of the development of the nervous system. Disruption of neuronal migration can lead to brain abnormalities associated with neurodevelopmental disorders, which are characterized by behavioral deficits. However, the underlying mechanisms by which abnormal neuronal migration and positioning generate defects in circuit organization and function are poorly understood. Here, we employed the migration of facial branchiomotor (FBM) neurons as a model to investigate the consequences of defective neuronal migration on the organization and functional outputs of the motor circuit. In this report, we use the word “circuit” to specifically refer to the connection between the branchiomotor neurons and their jaw muscle targets. While visual, olfactory, gustatory and hypothalamic inputs to the FBM and other branchiomotor neurons are essential elements of the branchiomotor circuit controlling jaw movement, the roles of these neural components have not been addressed here. Our analysis of the FBM migration mutant *off-limits* (*olt*) revealed subtle structural and functional deficits in the circuits controlling jaw movement. Our work indicates that while neuronal circuit function is largely resilient to changes in positioning of component neurons, there is potential for disrupting functional (behavioral) outputs that impact health and survival.

### Developmental Changes in Jaw Movement Behavior May Be Pre-synaptically Driven

Between 3 and 5 dpf, the frequency of jaw movement (gape) and its magnitude increased sharply from a very low level and remained elevated through 9 dpf. This time course of gape acquisition coincides with the time when zebrafish larvae start actively foraging for food (Budick and O’Malley, 2000; Carrillo and McHenry, 2016). While the motor output of the branchiomotor circuitry is established and becomes robust between 5 and 7 dpf, the branchiomotor axons have already extended on to the jaw muscles (Higashijima et al., 2000) and assembled neuromuscular junctions (NMJs) by 3 dpf. The lag between NMJ assembly and the onset of jaw movement may reflect the occurrence of additional events needed for the maturation of the circuit. These events can include changes in or further elaboration of the NMJs, arrival of inputs to the branchiomotor neurons from other brain regions, and acquisition of specific electrical properties by the motor neurons as a part of their differentiation process. Our data strongly support a role for changes in NMJs between branchiomotor axons and jaw muscles (Figure 2), but other sources for the maturation cannot be ruled out. For example, the spontaneous and coordinated activities of zebrafish tectal neurons between 2 and 4 dpf, when the visual system is becoming functional (Pietri et al., 2017), are driven in part by changes in their dendritic arborization and neurotransmitter identity (Boulanger-Weill et al., 2017). In another example of circuit maturation, the onset of robust activity in the respiratory circuits controlling breathing in mice between embryonic day 16 (E16) and E18 is driven by acquisition of neuromodulatory inputs and inputs from other rhythmic centers (Viemari et al., 2003). While the roles of neuromodulation and other inputs in circuit maturation can be readily studied by pharmacological manipulation in *in vitro* preparations, these approaches are not feasible for examining branchiomotor circuit maturation driving jaw movement in zebrafish. However, imaging of neuronal activity and *trans*-synaptic labeling to identify inputs from other brain regions can be employed to identify potential maturation mechanisms in addition to the NMJ changes that we have identified.

Our data suggest that the developmental onset of jaw movements between 3 and 5 dpf is a prerequisite for larvae to forage and capture food from 5 dpf. But these jaw movements could potentially represent buccal movements, which also increase between 3 and 9 dpf (Jonz and Nurse, 2005). However, a comparison of ventilation frequencies (generated by buccal movement) in 3–9 dpf larvae under normoxic and hypoxic conditions to 3–9 dpf gape frequencies reveals conflicts. First, the ventilation frequency is ∼0 in 6 dpf larvae under normoxia, whereas gape frequency at 5 dpf (mean ∼0.8 Hz) is closer to the ventilation frequency under hypoxia. On the other hand, gape frequency increases ∼33% from 5 to 7 dpf, whereas ventilation frequency under hypoxia increases ∼250% from 6 to 7 dpf (Jonz and Nurse, 2005). Therefore, developmental changes in gape frequency do not correspond to changes in ventilation frequencies either under normoxia or hypoxia. Hence, while larvae in our experimental conditions may experience hypoxia, the developmental maturation of gape follows a different trajectory than maturation of ventilation – the latter appears to result from the innervation of oxygen-sensing neuroepithelial cells in the gill filaments (Jonz and Nurse, 2005), whereas the former could result from pre-synaptic changes at the jaw NMJs (Figure 2).

### Defective FBM Neuron Position in *olt* Mutants Is Correlated With a Significant Functional Deficit in the Motor Circuit

We have documented two defective behavioral phenotypes in *olt* mutants that represent functional outputs of the branchiomotor circuit. First, gape (jaw opening) frequency is significantly reduced in mutants. Second, *olt* mutants eat less than their wildtype siblings. These data are the first demonstration of a defective functional output correlated with abnormal positioning of FBM neurons. However, we have not established a causal link between the two, and the observed functional defects may result from the loss of *fzd3a* function in various tissues rather than from the mispositioning of FBM neurons.

Breathing rhythms in mouse embryos, a motor output driven in part by the e-pF neurons that are immediately adjacent to the FBM neurons (Thoby-Brisson et al., 2009), are not disrupted in *Looptail* mutants where FBM neurons fail to migrate out of r4 (Thoby-Brisson et al., 2012). Even though e-pF neurons are significantly displaced in *Looptail* mutants due to the mispositioning of FBM neurons, the output of the respiratory motor circuit was largely unaffected, suggesting that functional outputs of circuits that are critical for survival may be resistant to changes in neuronal position (Thoby-Brisson et al., 2012). In contrast, our data suggest that larval jaw movement, a motor output that is necessary for food intake and survival, can be modified by changes in FBM neuron position. Interestingly, while the lower frequency of jaw movements is correlated with reduced food intake in *olt* mutants, it is not clear whether this feeding defect impacts survival since homozygous mutant *olt* larvae can grow into viable and fertile adults in a favorable lab environment with plentiful food and lack of competition from wildtype siblings. Further analysis is needed to evaluate whether food intake deficits measured in our assay translate to reduced survival in challenging environments that incorporate competition for limited food.

McArthur and Fetcho (2017) performed extensive analysis of *landlocked* (*llk*) and *prickle1b* mutants, both of which exhibit similar FBM neuron migration defects as *olt* mutants. Their studies did not reveal any changes in branchiomotor neuronal organization or branchiomotor circuit output, indicating that this motor circuit is resilient to changes in neuronal position, perhaps through compensatory mechanisms (McArthur and Fetcho, 2017). Furthermore, while this study analyzed and found no effect on opercular movement, a behavior driven by an FBM neuron-innervated muscle, one cannot rule out deficits in jaw muscle innervation and movement in *llk* mutants, as seen in *olt* mutants. Indeed, preliminary analyses indicate that gape frequency and food intake are reduced in *llk* mutants (Asante, E. and Chandrasekhar, A., unpublished data). Detailed analysis is needed to determine whether the behavioral deficits in *llk* mutants are distinct and different from those in *olt* mutants, and whether these are accompanied by changes in branchiomotor axon morphologies and NMJ structures.

There are several examples of human neuronal migration disorders (NMDs) and mouse NMD models where mispositioning of neurons is associated with behavioral deficits (Moffat et al., 2015). Aberrant migration of pyramidal projection neurons, or cortical interneurons and hippocampal neurons are associated with a broad range of cognitive and motor deficits seen in autism and schizophrenia, respectively (Moffat et al., 2015; Muraki and Tanigaki, 2015; Pan et al., 2019). Given the complex etiology of these disorders, it remains challenging to establish direct mechanistic links between mispositioned cortical or hippocampal neurons and specific behavioral deficits. Because of the relatively simple organization of the branchiomotor circuit mediating jaw movements, our ongoing studies will facilitate the elucidation of the molecular and cellular consequences of defective neuronal migration (and positioning) on circuit output.

### Are Branchiomotor Axon and Activity Defects in *olt* Mutants a Consequence of FBM Neuron Mispositioning?

Our data indicate that there are subtle defects in branchiomotor axon fasciculation and FBM neuron activity in 7 dpf *olt* mutants (Figures 5, 7) concomitant with reduced jaw movements. While we interpret these defects to be the consequence of FBM neurons being mislocated in r4 due to their failure to migrate caudally, we cannot rule out that these defects arise independently of FBM neuron mispositioning. Since *fzd3a*, the PCP gene inactivated in *olt* mutants, is broadly expressed in neural and non-neural tissues (Wada et al., 2006), and loss of *Fzd3* has been shown to disrupt the growth of several axon tracts in the developing mouse brain (Wang et al., 2002; Wang, 2006; Hua et al., 2014), FBM axon defects can be expected in *olt* mutants. Interestingly, most of the defasciculation defects in *olt* mutants are found on the ih and hh muscles that are innervated by FBM axons, suggesting the defasciculation phenotype may be preferentially associated with axons extended by the mispositioned motor neurons. Moreover, other aspects of axon outgrowth like length and branching from FBM and trigeminal axons are unaffected indicating that there are no widespread defects in neuronal development in *olt* mutants, and consistent with the normal development of several axon pathways in *Fzd3* mutant mice (Hua et al., 2014). The reduced frequency of Ca^2+^ events in *olt* mutants could be the consequence of the mispositioned FBM neurons expressing an altered repertoire of ion channels or receiving an aberrant combination of synaptic inputs from other brain regions. Alternatively, loss of *fzd3a* may lead to dysregulation of the non-canonical Wnt/Calcium pathway (Slusarski and Pelegri, 2007), resulting in altered Ca^2+^ dynamics within FBM neurons. Cell autonomous and non-autonomous effects of *fzd3a* loss on FBM neurons can be addressed by expression profiling analysis, as well as by identifying neuronal inputs from other brain regions.

The concomitant decreases in jaw movement (gape frequency) and Ca^2+^ event frequency in *olt* mutants may suggest a direct link between putative firing of FBM neurons and contraction of the jaw muscles they innervate. Indeed, the firing patterns of FBM neurons and opercular movements overlap in frequency (McArthur and Fetcho, 2017). However, the observed Ca^2+^ event frequency (∼0.02 Hz; Figure 7) is roughly 50-fold lower than the gape frequency (∼1 Hz; Figure 1) at 7 dpf. This could be a technical issue, since we used GCaMP6s to record Ca^2+^ events, rather than GCaMP6f (McArthur and Fetcho, 2017), resulting in a failure to detect spikes associated with FBM neuron firing (action potentials). Nevertheless, our data have established a link between activity changes within FBM neurons and the jaw movement output of the branchiomotor circuit. The modulation of this link by changes at the jaw NMJs and by regulatory inputs to the FBM neurons from other brain regions needs further study.

## Conclusion

Our analysis of jaw movement defects in *olt* mutants suggests that a functional output of the branchiomotor circuit is impacted by the mispositioning of one of the motor neuron populations driving the behavior. We also document changes in circuit structure and activity that could underlie the alteration in the motor output. In the future, we will further address the link between neuronal mispositioning and motor output by employing a variety of conditions to systematically vary FBM neuron position within the zebrafish hindbrain and evaluating their impacts on jaw movement and food intake, behavioral outputs of the branchiomotor circuit that are essential for survival.

## Data Availability Statement

The raw data supporting the conclusions of this article will be made available by the authors, without undue reservation.

## Ethics Statement

The animal study was reviewed and approved by Animal Care and Use Committee, University of Missouri.

## Author Contributions

EA designed and performed all experiments except those indicated, analyzed the data, and co-wrote the manuscript. DH generated the *Tg(zCREST1:GCaMP6s)* line and helped with the GCaMP imaging studies. SG and VS performed the swimming analysis of *off-limits* larvae using the Noldus system. YMK and NA-S wrote and validated the gape analysis software, transferred the code to MacOS, and were supervised by KP. All authors commented on and contributed to manuscript revisions. KP and AC obtained funding. AC conceived of the project, supervised the experiments, analyzed the data, and co-wrote the manuscript.

## Conflict of Interest

The authors declare that the research was conducted in the absence of any commercial or financial relationships that could be construed as a potential conflict of interest.

## Supplementary Material

Supplementary Figure 1 System for imaging jaw movement and processing steps in motion analysis software. Flowchart of high-throughput video imaging pipeline for larval zebrafish jaw movement and gape frequency analysis using motion estimation and fusion. This computerized motion-based analysis tool produces a digital response for gape or mouth opening of zebrafish microscopy videos in different development stages within a Region of Interest (ROI). ROI is manually specified in the first frame (red box). The system relies on multiple motion-based approaches [optical flow + Background subtraction (BG)] of jaw movements followed by a robust fusion of motion fields. Optical flow is used to obtain four directional motion maps of the cropped region using the optical flow vectors (*u*, *v*), and BG is used to obtain a precise region of motion for the jaw and filter out responses generated by global animal movement within the ROI. The algorithmic fusion fuses BG mask with the motion magnitude map of the flow field within the jaw’s desired direction (southwest) and produces the gape response. The software was validated by testing it on 24 videos (2,400 video frames), with an average accuracy of 98.7 and 1.2 standard deviation, making it robust enough to analyze other videos generated in the study.

Supplementary Figure 2 Morphometric analysis of the heads of 5 and 7 dpf larvae. All measurements were made on single frames captured from time-lapse recordings of laterally mounted larvae. **(A)** Head height was measured at the level of the epiphysis to the base of the lower jaw. The eye diameter was measured at the same rostrocaudal level as the head height. **(B)** The ratio of the eye diameter to head height was calculated in 5 and 7 dpf larvae. There were no differences between the two ages for *Tg(isl1:GFP)* larvae, as well as *olt* mutants and wildtype siblings. Number of larvae is indicated in parenthesis.

Supplementary Figure 3 Measurement of volumes of presynaptic and postsynaptic structures at the NMJs. Flowchart of 3D imaging of NMJ imaging and analysis. In the image analysis pipeline, *Z*-stack data files were processed and a new *z*-stack in 3D was created in LAS X. The newly created 2-channel *z*-stack stack was opened in the 3D mode in the multi channel. This created two channels “NMJ” and “Muscles” **(Left upper and lower panels)**. Channels were thresholded using the “binary” image (original *z*-stack image) in a separate window view to adjust the threshold value to the point when all objects in the channels are accurately and precisely defined. The “Draw Straight Lines” method was selected and the lines between the muscle’s segments (ima/p, amR, amL, ihR, ihL, hhR, and hhL) were color coded and divided into muscles in the “Binary Image Editing” window only in Muscles channel **(Middle lower panel)**. The “Reference Mask” was marked in the Muscles channel and “Count, Intensity, Area mode” was marked in the NMJ channel **(Middle upper panel)**. The right upper panel shows the binary image of the NMJ channel **(left side)**, and the NMJ channel with overlaid measurement reference mask **(right side)**. The Results table with the measurements and summary of all objects within the measured muscles were exported and the new 3D image was saved as a “configuration in 3D” file.

Supplementary Figure 4 Gape frequencies for distributed and clustered patterns are reduced in *olt* mutants. **(A)** Within the samples of distributed gape patterns, gape frequencies in 5, 7, and 9 dpf *olt* mutant larvae were significantly reduced compared to wildtype siblings. **(B)** Within the samples of clustered gape patterns, gape frequencies in 7 and 9 dpf *olt* mutant larvae were significantly reduced compared to wildtype siblings. Statistical analysis was performed with a two-tailed student *t*-test, ^∗∗^*p* < 0.001. Number of larvae in parenthesis.

Supplementary Figure 5 Measurement of motor axon lengths and branch numbers. Flowchart of axon imaging and analysis using *Tg (zCREST1:mRFP)* larvae. Confocal image stacks were 3D rendered, allowed for manual tracing of trigeminal axon and FBMN axons. The tracing and measurement processes were performed in LAS-X using the “polygon” tool, starting at the hindbrain exit points for both motor axon fascicles. At every step of tracing axons and branches, the 3D images were rotated and viewed at different angles, and the polygon tool was adjusted to ensure that it was on the surface of the axon. Data were exported and traced images were saved as configuration in 3D.

Supplementary Figure 6 Axon fascicles on ih muscle are more numerous and thinner in *olt* mutants. **(A,B)** Ventral view with anterior to the left of the jaw musculature in 5 dpf *Tg(zCREST1:mRFP);Tg(α-actin:GFP)* larvae showing motor axons (red) and jaw and gill muscles (green). The numbered lines 1–6 indicate the positions of the virtual sections along the left and right ih muscles where the red fluorescence intensities corresponding to axon fascicles were plotted to count fascicles and measure their thickness. **(C)** The number of axon fascicles was significantly higher in *olt* mutants. **(F)** The axon fascicles were significantly thinner in *olt* mutants.

Supplementary Figure 7 Facial branchiomotor neurons are less active in *olt* mutants. Re-analysis of FBM neuron activity in a subset of samples that were used for analysis of activity in trigeminal motor neurons in Figure 7. **(A,B)** Representative GCaMP6s Δ*F*/*F* traces for two wildtype **(A)** and two *olt* mutant **(B)** larvae. Due to the broad peaks, thresholding-based event counting was not used. Instead, Ca^2+^ events (asterisks) were manually identified by two observers independently by viewing the recordings. **(C)** The frequency of Ca^2+^ events in FBM neurons was significantly lower in *olt* mutants compared to wildtype siblings as seen in Figure 7. Number of larvae in parenthesis.

Supplementary Movie 1 Lateral view of a 3 dpf larva, 18 frames per second, 600 frames. Speeded up 1.65×.

Supplementary Movie 2 Lateral view of a 5 dpf larva, 18 frames per second, 600 frames. Speeded up 1.65×.

Supplementary Movie 3 Lateral view of a 7 dpf larva, 18 frames per second, 600 frames. Real time speed.

Supplementary Movie 4 Lateral view of a 7 dpf wildtype sibling larva, 18 frames per second, 600 frames. Speeded up 1.65×.

Supplementary Movie 5 Lateral view of a 7 dpf *olt* mutant larva, 18 frames per second, 600 frames. Speeded up 1.65×.

Supplementary Movie 6 Rotation of image stacks to reveal the 3-dimensional morphologies of the motor axons **(top left panel)** and their terminations on the jaw muscles **(bottom panel)**. The top right panel shows the polygon tracing method used to measure axon lengths and branch numbers.

Supplementary Movie 7 Dorsal view of the hindbrain of a 7 dpf wildtype sibling. Images of changes in Ca^2+^ levels in branchiomotor neurons were obtained in the GFP channel at 2 frames per second for 5 min. Speeded up 12.5×.

Supplementary Movie 8 Dorsal view of the hindbrain of a 7 dpf *olt* mutant. Images of changes in Ca^2+^ levels in branchiomotor neurons were obtained in the GFP channel at 2 frames per second for 5 min. Speeded up 12.5×.
