# Supplementary figures and images for: Defective Neuronal Positioning Correlates With Aberrant Motor Circuit Function in Zebrafish
Source: Front Neural Circuits. 2021 Jun 24;15:690475. doi: 10.3389/fncir.2021.690475 (PMC8265374; doi:10.3389/fncir.2021.690475)

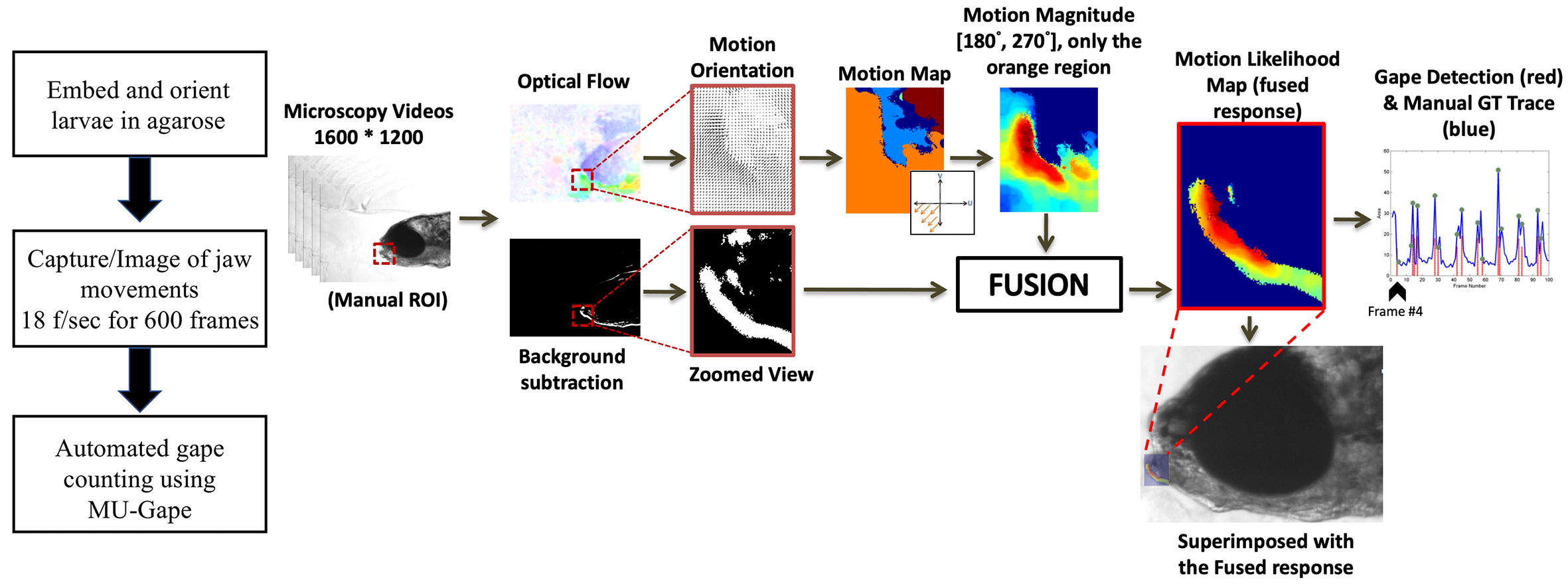

Supplement: Supplementary Figure 1 — System for imaging jaw movement and processing steps in motion analysis software. Flowchart of high-throughput video imaging pipeline for larval zebrafish jaw movement and gape frequency analysis using motion estimation and fusion. This computerized motion-based analysis tool produces a digital response for gape or mouth opening of zebrafish microscopy videos in different development stages within a Region of Interest (ROI). ROI is manually specified in the first frame (red box). The system relies on multiple motion-based approaches [optical flow + Background subtraction (BG)] of jaw movements followed by a robust fusion of motion fields. Optical flow is used to obtain four directional motion maps of the cropped region using the optical flow vectors (u, v), and BG is used to obtain a precise region of motion for the jaw and filter out responses generated by global animal movement within the ROI. The algorithmic fusion fuses BG mask with the motion magnitude map of the flow field within the jaw’s desired direction (southwest) and produces the gape response. The software was validated by testing it on 24 videos (2,400 video frames), with an average accuracy of 98.7 and 1.2 standard deviation, making it robust enough to analyze other videos generated in the study. [file Image_1.tif]

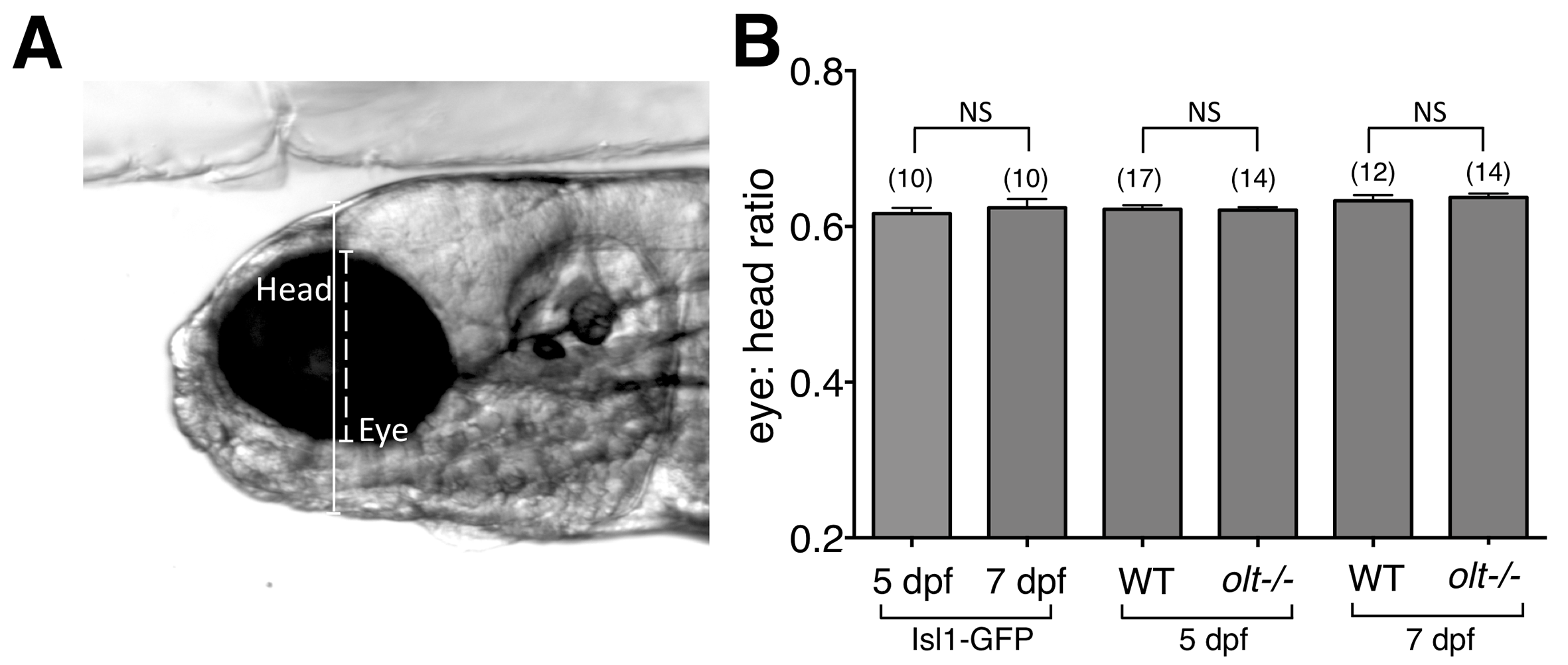

Supplement: Supplementary Figure 2 — Morphometric analysis of the heads of 5 and 7 dpf larvae. All measurements were made on single frames captured from time-lapse recordings of laterally mounted larvae. (A) Head height was measured at the level of the epiphysis to the base of the lower jaw. The eye diameter was measured at the same rostrocaudal level as the head height. (B) The ratio of the eye diameter to head height was calculated in 5 and 7 dpf larvae. There were no differences between the two ages for Tg(isl1:GFP) larvae, as well as olt mutants and wildtype siblings. Number of larvae is indicated in parenthesis. [file Image_2.TIF]

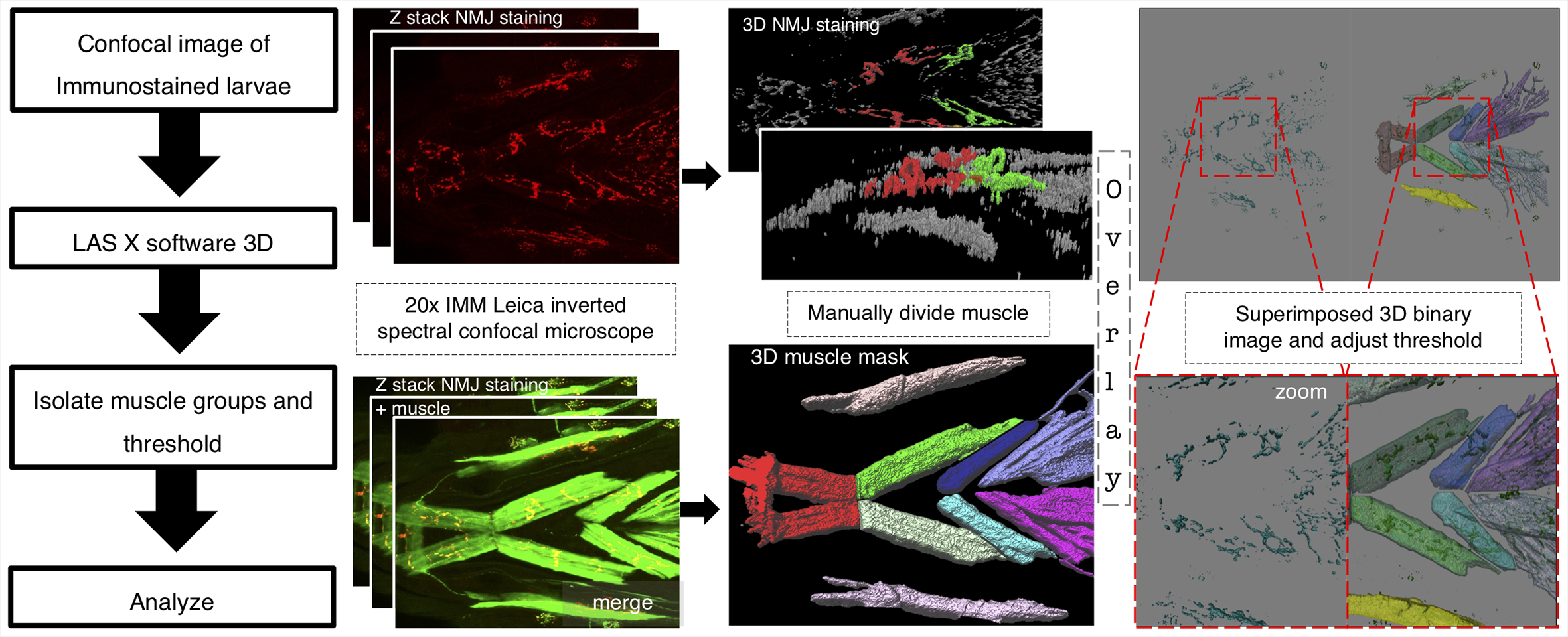

Supplement: Supplementary Figure 3 — Measurement of volumes of presynaptic and postsynaptic structures at the NMJs. Flowchart of 3D imaging of NMJ imaging and analysis. In the image analysis pipeline, Z-stack data files were processed and a new z-stack in 3D was created in LAS X. The newly created 2-channel z-stack stack was opened in the 3D mode in the multi channel. This created two channels “NMJ” and “Muscles” (Left upper and lower panels). Channels were thresholded using the “binary” image (original z-stack image) in a separate window view to adjust the threshold value to the point when all objects in the channels are accurately and precisely defined. The “Draw Straight Lines” method was selected and the lines between the muscle’s segments (ima/p, amR, amL, ihR, ihL, hhR, and hhL) were color coded and divided into muscles in the “Binary Image Editing” window only in Muscles channel (Middle lower panel). The “Reference Mask” was marked in the Muscles channel and “Count, Intensity, Area mode” was marked in the NMJ channel (Middle upper panel). The right upper panel shows the binary image of the NMJ channel (left side), and the NMJ channel with overlaid measurement reference mask (right side). The Results table with the measurements and summary of all objects within the measured muscles were exported and the new 3D image was saved as a “configuration in 3D” file. [file Image_3.TIF]

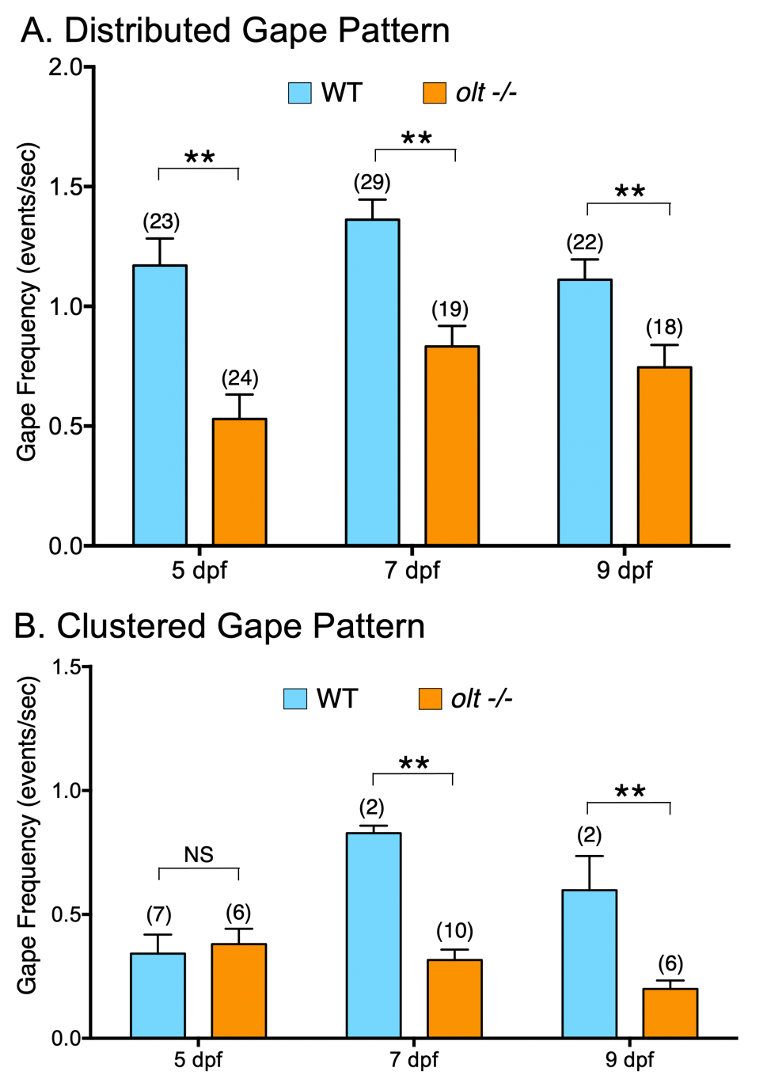

Supplement: Supplementary Figure 4 — Gape frequencies for distributed and clustered patterns are reduced in olt mutants. (A) Within the samples of distributed gape patterns, gape frequencies in 5, 7, and 9 dpf olt mutant larvae were significantly reduced compared to wildtype siblings. (B) Within the samples of clustered gape patterns, gape frequencies in 7 and 9 dpf olt mutant larvae were significantly reduced compared to wildtype siblings. Statistical analysis was performed with a two-tailed student t-test, ∗∗p < 0.001. Number of larvae in parenthesis. [file Image_4.TIF]

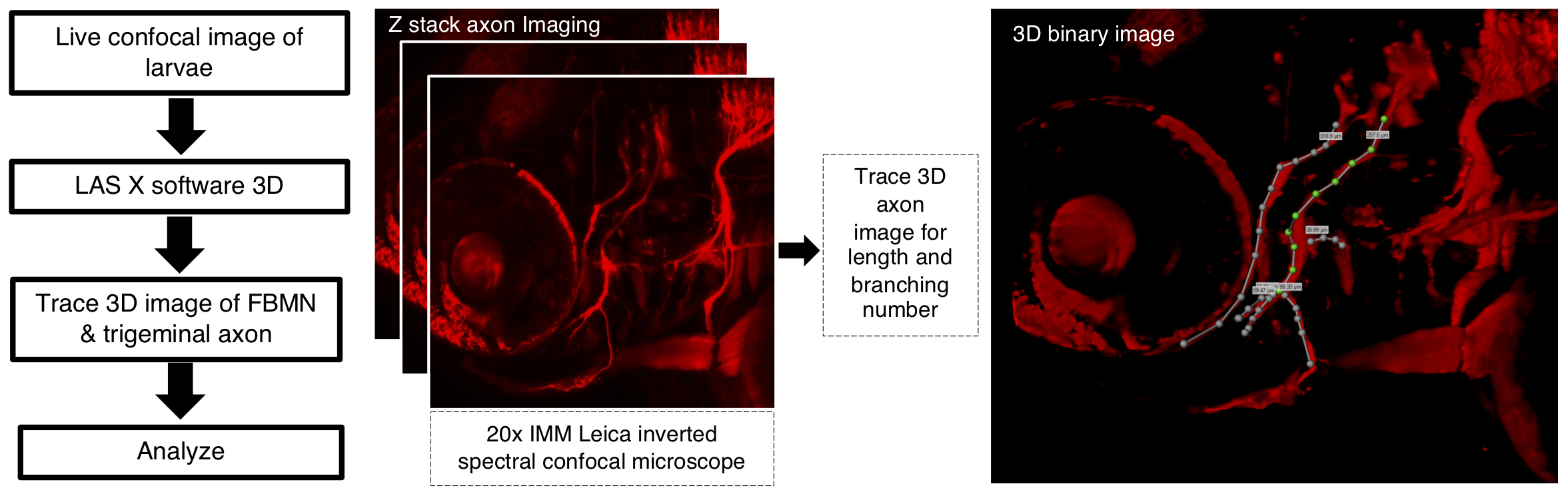

Supplement: Supplementary Figure 5 — Measurement of motor axon lengths and branch numbers. Flowchart of axon imaging and analysis using Tg (zCREST1:mRFP) larvae. Confocal image stacks were 3D rendered, allowed for manual tracing of trigeminal axon and FBMN axons. The tracing and measurement processes were performed in LAS-X using the “polygon” tool, starting at the hindbrain exit points for both motor axon fascicles. At every step of tracing axons and branches, the 3D images were rotated and viewed at different angles, and the polygon tool was adjusted to ensure that it was on the surface of the axon. Data were exported and traced images were saved as configuration in 3D. [file Image_5.TIF]

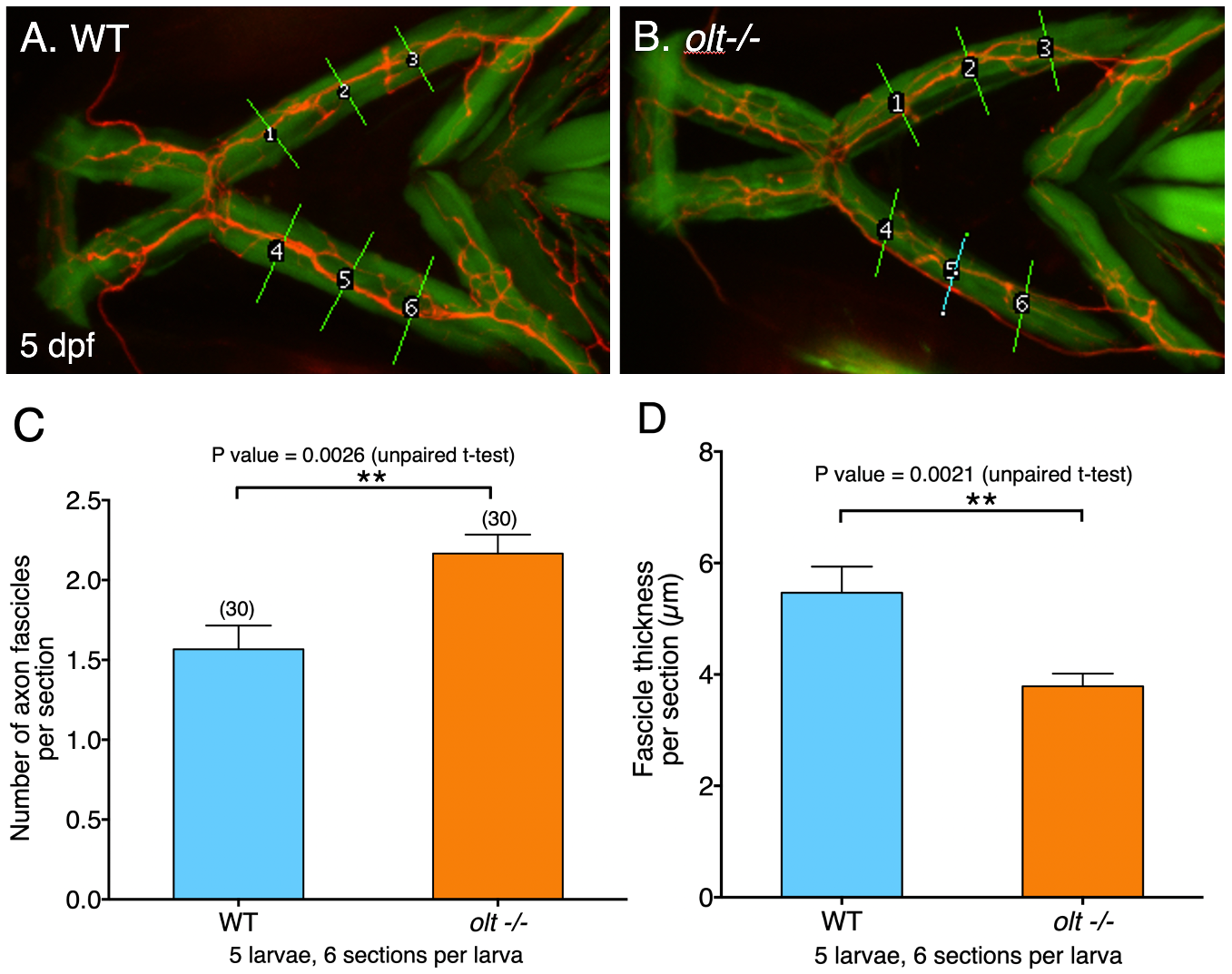

Supplement: Supplementary Figure 6 — Axon fascicles on ih muscle are more numerous and thinner in olt mutants. (A,B) Ventral view with anterior to the left of the jaw musculature in 5 dpf Tg(zCREST1:mRFP);Tg(α-actin:GFP) larvae showing motor axons (red) and jaw and gill muscles (green). The numbered lines 1–6 indicate the positions of the virtual sections along the left and right ih muscles where the red fluorescence intensities corresponding to axon fascicles were plotted to count fascicles and measure their thickness. (C) The number of axon fascicles was significantly higher in olt mutants. (F) The axon fascicles were significantly thinner in olt mutants. [file Image_6.TIF]

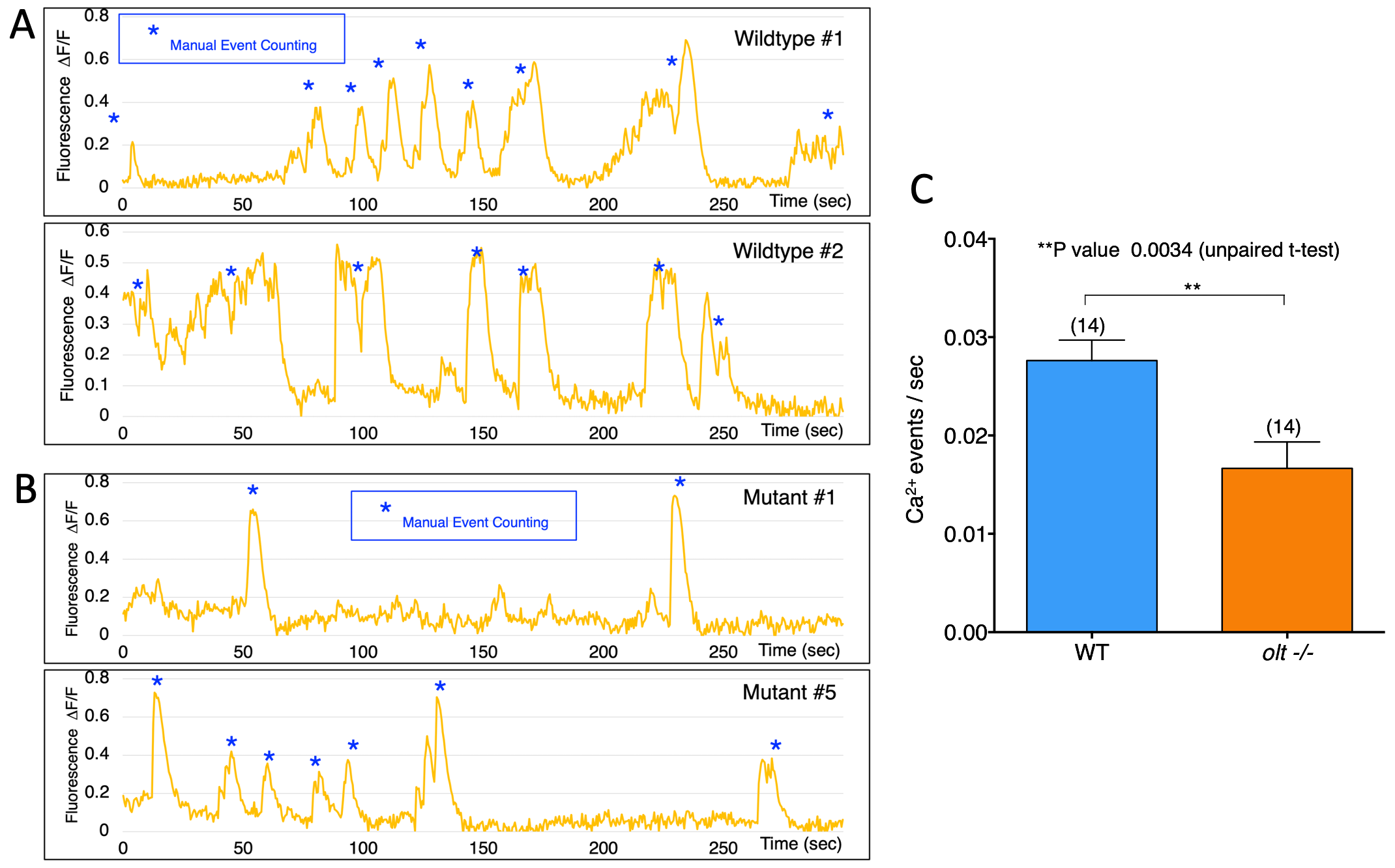

Supplement: Supplementary Figure 7 — Facial branchiomotor neurons are less active in olt mutants. Re-analysis of FBM neuron activity in a subset of samples that were used for analysis of activity in trigeminal motor neurons in Figure 7. (A,B) Representative GCaMP6s ΔF/F traces for two wildtype (A) and two olt mutant (B) larvae. Due to the broad peaks, thresholding-based event counting was not used. Instead, Ca2+ events (asterisks) were manually identified by two observers independently by viewing the recordings. (C) The frequency of Ca2+ events in FBM neurons was significantly lower in olt mutants compared to wildtype siblings as seen in Figure 7. Number of larvae in parenthesis. [file Image_7.TIF]
